# Exploring the interplay between natural and intersexual selection on the evolution of a cognitive trait

**DOI:** 10.1002/ece3.9066

**Published:** 2022-07-04

**Authors:** Marie Barou‐Dagues, Frédérique Dubois

**Affiliations:** ^1^ Centre d'Etudes Biologiques de Chizé, CNRS‐ULR Villiers en Bois France; ^2^ Département des Sciences Biologiques Université de Montréal Montréal Quebec Canada

**Keywords:** cognitive performance, intersexual selection, mate choice, problem‐solving ability, provisioning ability

## Abstract

There has been an increased focus on the role of natural and sexual selection in shaping cognitive abilities, but the importance of the interaction between both forces remains largely unknown. Intersexual selection through female mate choice might be an important driver of the evolution of cognitive traits, especially in monogamous species, where females may obtain direct fitness benefits by choosing mates with better cognitive abilities. However, the importance given by female to male cognitive traits might vary among species and/or populations according to their life‐history traits and ecology. To disentangle the effects of natural and sexual selection, here we use an agent‐based simulation model and compare the model's predictions when females mate with the first randomly encountered male (i.e., under natural selection) versus when they choose among males based on their cognitive trait values (i.e., under natural and intersexual selection). Males and females are characterized, respectively, by their problem‐solving ability and assessment strategy. At each generation, agents go through (1) a choosing phase during which females assess the cognitive abilities of potential mates until eventually finding an acceptable one and (2) a reproductive phase during which all males compete for limited resources that are exploited at a rate, which depends on their cognitive abilities. Because males provide paternal care, the foraging success of mated males determines the breeding success of the pair through its effect on nestling provisioning efficiency. The model predicts that intersexual selection plays a major role in most ecological conditions, by either reinforcing or acting against the effect of natural selection. The latter case occurs under harsh environmental conditions, where intersexual selection contributes to maintaining cognitive diversity. Our findings thus demonstrate the importance of considering the interaction between both selective forces and highlight the need to build a conceptual framework to target relevant cognitive traits.

## Graphical abstract

We developed an agent‐based simulation model to investigate the role of natural and intersexual selection through female mate choice on the evolution of a cognitive trait in monogamous species. Here, we predict that intersexual selection should play a major role in most ecological conditions, by either reinforcing or acting against the effect of natural selection. Specifically, female mate choice might favour the rapid evolution of high cognitive abilities in favourable environments but might contribute to maintaining cognitive diversity in harsher ones.

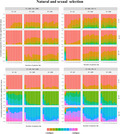

## 1 INTRODUCTION

Cognition is defined as the neural processes by which animals sense, process, retain, and act on the available information (Shettleworth, 2001). As such, it plays an important role in mediating how animals behave and interact with their environment and may have important fitness consequences (Morand‐Ferron & Quinn, 2015). The most direct evidence for a link between cognitive abilities and fitness benefits comes from studies on wild populations where individuals that performed better on cognitive tasks were more successful foragers (Raine & Chittka, 2008), survived better (Cole et al., 2012), had more mating partners (Keagy et al., 2009), produced more offspring (Ashton et al., 2018; Cauchard et al., 2013; Cole et al., 2012), and had higher provisioning and fledging rate (Cauchard et al., 2017; Preiszner et al., 2017; Wetzel, 2017). Given that cognitive abilities are, to some extent, heritable (Croston et al., 2015; Hopkins et al., 2014; Langley, van Horik, et al., 2020; Navas González et al., 2019 but see Quinn et al., 2016), these findings indicate that cognitive traits might evolve by natural and/or sexual selection. There has been an increased focus on the role of natural (e.g., Morand‐Ferron & Quinn, 2015; Rowe & Healy, 2014) and sexual (e.g., Boogert et al., 2011) selection in shaping cognitive abilities. However, the importance of the interplay between both forces remains largely unexplored.

Yet it is acknowledged that natural and sexual selection may interact to shape the evolution of phenotypic (Castillo & Núñez‐Farfán, 2008; Jiménez‐Arcos et al., 2017; Ryder et al., 2012) and signaling (Ríos‐Chelén, 2009) traits, hence the importance of considering both selective forces. Intra and intersexual selection could actually improve male cognitive abilities (e.g., Arden et al., 2009; Boogert et al., 2011; Garamszegi et al., 2005; Schillaci, 2006). For instance, intra‐sexual selection might enhance male cognition if males with greater cognitive abilities are better able to locate and discriminate among mates. Accordingly, studies based on experimental evolution revealed that males of polygamous lines facing high levels of sexual competition had better performance on certain cognitive tasks compared with males of monogamous lines facing no competition (Baur et al., 2019; Hollis & Kawecki, 2014). Specifically, intra‐sexual selection improved cognitive abilities that affected the capacity of males to discriminate between receptive and unreceptive females but not their learning speed. Intersexual selection might also be a driver of cognitive abilities if females gain direct and/or indirect fitness benefits by choosing males with cognitive abilities that are, for instance, correlated with their foraging success, predator avoidance, or parental care capacity (Rosenthal, 2017; Snowberg & Benkman, 2009; Wetzel, 2017). There are lines of evidence that intersexual selection may indeed shape the evolution of male cognition (Boogert et al., 2011; Branch et al., 2019; Cauchard et al., 2013; Cole et al., 2012; Keagy et al., 2009, 2011; Minter et al., 2017; Preiszner et al., 2017; Shaw et al., 2019; Shohet & Watt, 2009; Wetzel, 2017). For instance, a few studies found indirect support that females would prefer males with greater cognitive abilities, by demonstrating that they base their mate choice decision on secondary sexual characters (e.g., male song and plumage coloration) that correlate with better cognitive skills (Cauchard et al., 2017; Howell et al., 2020). A more direct support for this hypothesis comes from an experiment in which females increased their preference towards initially nonpreferred males, after they had observed that these males (but not their rivals) could solve a specific problem (Chen et al., 2019). Yet, other studies found no relationship between male cognitive performance measures and mating success (Chantal et al., 2016; Isden et al., 2013; Preiszner et al., 2017).

The discrepancy among studies strongly suggests that the importance given by females to male cognitive traits would vary among species and/or populations according to their life‐history traits and ecology, and among females according to their own characteristics (Barou‐Dagues & Dubois, 2022). For instance, good problem‐solving skills, although sometimes being problematic measures of cognitive abilities (Van Horik & Madden, 2016), are related to higher provisioning rates and offspring survival and so may be important determinants of parental care (Wetzel, 2017). As such, they might be the principal targets of mate choice in monogamous species with bi‐parental care. The importance is given by females to male cognitive skills that correlate with provisioning effort, however, should depend, among other factors, on the quality, quantity, or distribution of available resources, and on the ability of females to accurately assess male cognitive traits and so on their mate assessment strategy. For instance, a recent study has demonstrated that some females make fast but inaccurate mate decisions while others make slow but accurate ones (Pauli & Linsdtrom, 2021). Furthermore, the effect of natural and sexual selection might interact as the ecological conditions experienced by individuals acting on the variance in male cognitive performance and, as such, determine the potential for intersexual selection within populations.

Here, we developed an agent‐based simulation model to explore the relative importance of natural and intersexual selection on (1) male cognitive traits that provide direct fitness benefits to females and (2) female assessment strategies determining the capacity to accurately assess male cognitive traits. Males and females were thus characterized, respectively, by their problem‐solving ability and strategy to reliably assess males' cognitive trait value, and we simulated the evolution of the male trait and female strategy over time under different ecological scenarios. A simulation is divided into two phases: (1) a choosing phase during which females assess the cognitive abilities of potential mates until eventually finding an acceptable one and (2) a reproductive phase during which all males compete for limited resources that are exploited at a rate, which depends on their cognitive abilities. Because males provide paternal care, the foraging success of mated males determines the breeding success of the pair through its effect on nestling provisioning efficiency. Each scenario (i.e., set of parameter values) is run when females mate with the first randomly encountered male (i.e., under natural selection alone) and when they can choose among males based on their cognitive trait values (i.e., under natural and intersexual selection) to be able to disentangle their respective effects. Predictions from the model indicate that intersexual selection through female mate choice should play a major role in most conditions, by either reinforcing or acting against the effect of natural selection under favorable or harsh environments, respectively. As such, we might expect female mate choice to contribute either to the rapid evolution of high cognitive abilities in favorable environments or to maintaining cognitive diversity in harsher ones.

## 2 AN AGENT‐BASED SIMULATION MODEL

**FIGURE 1:**
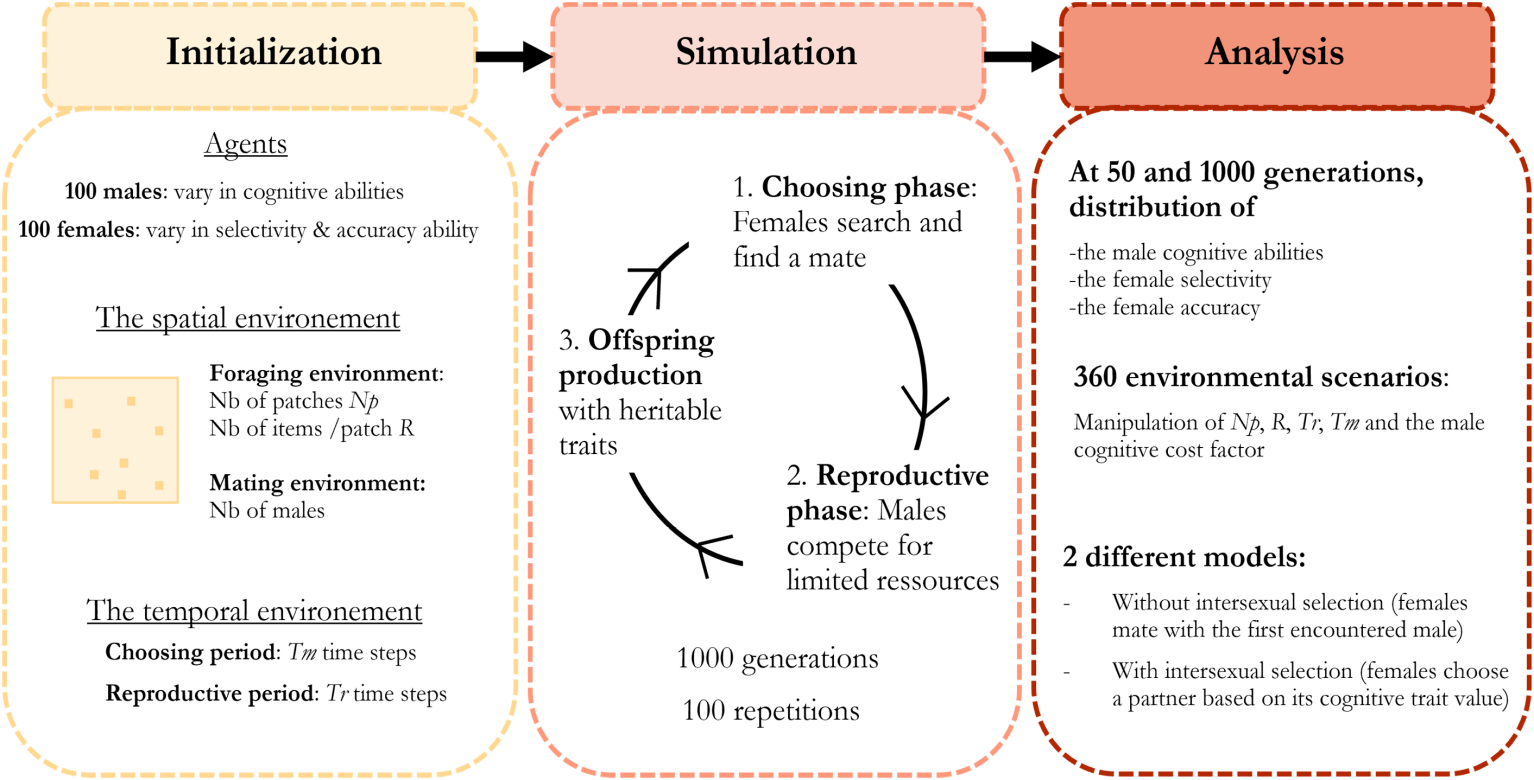
Overview of the model initialization, simulation processes, and analysis

All model parameters are presented in Table 1. A population contains a constant number of 100 males and 100 females that can move over a two‐dimensional grid containing 196 (14 × 14) territories. Each male *i* is characterized by a cognitive trait value (*I*_*i*_) ranging from 0 to 10, which represents the number of time steps required to solve a novel problem and access to food. This ability is especially relevant for species that need to remove obstacles, use tools, or innovate, as the faster they resolve the problem the faster they gain access to the food resource (e.g., Cole et al., 2011; Huebner et al., 2018). Thus, a low cognitive trait value represents males with good cognitive abilities while a high cognitive trait value represents males with poor cognitive abilities. Each female *j* is characterized by a mate assessment strategy that is determined by two state‐dependent variables representing, respectively, their capacity to accurately assess male cognitive trait value ($A_{j}$) ranging from 0 to 5 and their selectivity ($S_{j}$) ranging from 0 to 10. A simulation consists of 1000 consecutive generations, each of them divided into two phases: (1) a choosing phase during which females search for a breeding partner and (2) a reproductive phase during which all males compete for limited resources. The foraging success of males directly depends on their cognitive trait value and on the cost of cognition. Furthermore, we assume that only mated males provide parental care. As such, their foraging success determines the breeding success of the pair, through its effect on nestling provisioning efficiency (Figure 1, for more details about the code, see Supplementary material).

**TABLE 1:** Definition of the manipulated environmental and agent parameters. For each parameter, the symbol and the tested values are specified.

Environmental parameters	Symbol	Tested values
Number of food patches	*Np*	5, 25, 45, …, 185
Number of food items	*R*	10, 100, 200
Length of the reproductive phase (in time steps)	*Tr*	50, 200
Length of the choosing phase (in time steps)	*Tm*	5, 200
Agent parameters		
Cognition factor	∝	1, 6, 10

### 2.1 Choosing phase

At the beginning of the choosing phase, males and females, in turn, are randomly assigned to a unique location on the grid. Males stay on their assigned territory, while females can move among male territories during *Tm* time steps or until finding an acceptable mate using a random walk (i.e., only one random move per time step towards any male territory). When a female moves on the territory of a male that is not paired yet, she assesses the value of his cognitive trait during *A*_*j*_ time steps. We assume that females with a value of *A*_*j*_ equal to 5 (i.e., that spent 5 time units per male for mate assessment) perfectly assess male cognitive trait values while females with smaller values, obtain wrong estimates, the amount of error (ε) being inversely proportional to *A*_*j*_, such as: $\varepsilon =5-A_{j}$. Thus, the estimated value ($E_{i}$) corresponds to the exact trait value, to which we randomly add or subtract ε. Based on her estimate of the males' trait value and her minimum acceptance value (i.e., her selectivity *S*_*j*_), the female then either accepts to mate with the male (if $E_{i}\leq S_{j}$) or moves to another male territory. Specifically, the variable $S_{j}$ ranges from 0 to 10, females with a value of zero accepting to mate only with males with the highest cognitive trait value.

Hence, except for the indirect cost of making an assessment error, female choice is not costly. For the first generation, the accuracy and selectivity trait values of each female are randomly drawn from discrete uniform distributions with all values having the same probability of being picked. Subsequently, female offspring inherit their mothers' trait values.

### 2.2 Reproductive phase

During $T_{r}$ time steps (the duration of the reproductive phase), all males (i.e., mated and unmated) search for food patchily distributed that is used, in the case of mated males, to provision their young. More precisely, the environment contains above 196 territories; among them, $N_{F}$ contains *R* food items. At the beginning of a reproductive period, each male is randomly assigned to a territory and then can move from one territory to another using a random walk (i.e., one random move per time step in one of the four cardinal directions towards a territory). When a male moves on a territory that contains food, it must wait for *I*_*i*_ time steps (i.e., where *I*_*i*_ is the cognitive trait value also referred to as the problem‐solving ability) before it can start exploiting it. For the first generation, the cognitive trait value of each male is randomly drawn from a discrete uniform distribution with all values having the same probability of being picked. Subsequently, the male offspring inherit their fathers' trait value. Males suffer a cognitive cost that is inversely proportional to their cognitive trait value. Thus, each male *i* is characterized by its foraging success $W_{i}$ that is initialized to zero at the beginning of the reproductive phase and then incremented by one each time it gets one food item. At the end of the reproductive phase, we subtract from its success $W_{i}$ a cognitive cost $C_{i}=-\propto \left[10-I_{i}\right]$ that is more or less influenced by its problem‐solving ability *I*_*i*_, depending on the cognitive cost factor ∝ (with 1≤∝≤10, Table 1). Specifically, the cognitive cost *C*_*i*_ varies from 0 (i.e., for poorer problem solvers) to −10 (i.e., better problem solvers) when the cognitive cost factor ∝=1 but from 0 to −100 for poorer and better problem solvers, respectively, when ∝=10. We assume that each male can exploit only one food item at each time step and that a food patch can be simultaneously exploited by several males. In that case, each male that joins the food patch must wait the number of time steps associated with its cognitive trait value before it can get food. The number of food patches is kept constant throughout the reproductive phase. Therefore, once a food patch is depleted, it is immediately replaced by another one, whose location is randomly chosen among all unoccupied and empty territories.

At the end of the reproductive phase, we attribute each female a reproductive score ranging from 0 to 11. Unmated females have attributed a score of zero while the score attributed to mated females is proportional to the foraging success of their mating partner. Specifically, we attribute a reproductive score of zero to females whose partner had a foraging success equal to or less than zero. For the other females whose partner has a foraging success greater than 1, their score is incremented by 1 for every 10 units of foraging success with a maximum reproductive score of 11. Thus, all females whose partner has a foraging success equal to 100 food items or more obtain the same reproductive success. We used this threshold to homogenize the variance in reproductive success among simulations and across all environmental conditions where the total amount of food items available, and thus the male foraging success, can vary drastically. Thus, we can compare the reproductive success of pairs under favorable versus more challenging environmental conditions (e.g., when food is abundant or scarce). We assume that breeding pairs produce male and female offspring in equal proportion. In addition, population size is kept constant from one generation to the next and the population is completely renewed at each generation. Among all the offspring produced, therefore, we keep the proportion of trait values by randomly selecting 100 males and 100 females at the end of each mating period to constitute the next generation. To account for stochastic effects, the same simulation is run 100 times.

### 2.3 Analyses

In order to predict the relative importance of natural and sexual selection on the evolution of male cognition and female assessment strategies under different scenarios, we manipulated (i) the food distribution (i.e., *N*_*p*_ and R), (ii) the duration of the reproductive phase (i.e., *Tr*), (iii) the duration of the mating period (i.e., *Tm*) and iv) the cognitive cost *C*_*i*_ through manipulating the cognitive cost factor ∝, for a total of 360 sets of parameter values (see Table 1). By completely renewing the population at each generation, we imposed a very strong selective pressure on traits that are 100% heritable. Then, to follow trait diversity within populations, we only studied the evolution of cognitive traits after 50 generations. Note, however, that the predicted patterns remain unchanged if we look at the variance among populations after 1000 generations, once all populations have reached fixation (Figures 4 and 5; Appendix, Figures A3 and A4). Each set of parameter values was run when (1) females mated with the first randomly encountered male (i.e., under natural selection alone) and (2) when they could choose among males based on their cognitive trait values (i.e., under natural and sexual selection) to be able to disentangle the effects of natural and sexual selection. The simulation model was developed using the Python programming language, while the simulations were run on Canada compute servers (Calcul Quebec). All figures were generated in R studio (R Core Team, 2021; version 4.0.2).

## 3 RESULTS

### 3.1 Evolution of cognition under natural selection

**FIGURE 2:**
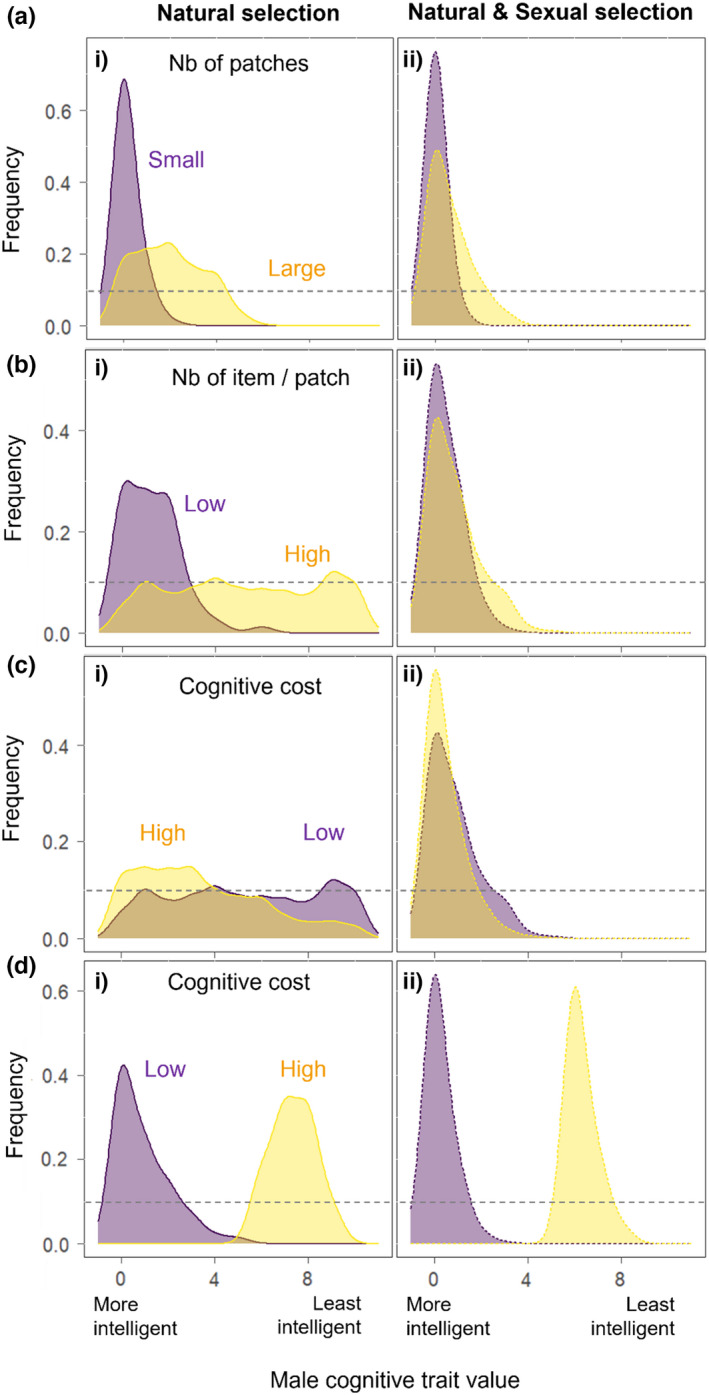
Effect of environmental parameters on the pooled distribution of male cognitive trait values at the 50th generation from 100 repetitions, under natural selection alone (left panels) or under natural and sexual selection (right panel). Low and high male cognitive trait values respectively mean that the males have good and low cognitive abilities. (a) *Tr* = 200, *Tm* = 200, *R* = 10, *α* = 1, and *Np* is manipulated (b) *Tr* = 200, *Tm* = 200, *Np* = 85, *α* = 1, and *R* is manipulated, (c) *Tr* = 200, *Tm* = 200, *R* = 100, *Np* = 85, and *α* is manipulated, (d) *Tr* = 50, *Tm* = 200, *R* = 100, *Np* = 85, and *α* is manipulated. The purple and yellow colors, respectively, represent low and high values of the manipulated factor, and the gray dashed line represent the initial distribution of the trait

The model predicts that when food resources are abundant and the reproductive phase is long, the strength of natural selection should be relatively weak and, as such, male initial cognitive diversity should be only slightly eroded (Figure 2Ai,Bi; Figure 4). Under such conditions, males can exploit a large amount of food items, and all achieve a high foraging success, irrespective of their cognitive ability. Increasing the cognitive cost, therefore, only slightly decreases male foraging success and the variance in foraging success between males. Specifically, cognitive diversity should be fully maintained when the cost of cognition is low because males all reach the maximum reproductive score. Yet, it should be slightly eroded in favor of males with better cognitive abilities when the cost of cognition is high (Figure 2Ci) because males with better cognitive abilities still reach the maximum reproductive score while the others obtain a slightly lower reproductive score. For that reason, the cost of cognition has generally a low impact on the female reproductive score and male cognitive diversity should mostly be maintained over time, regardless of the magnitude of the cost of cognition (Figure 2Ci). In contrast, the trade‐off between the cost and benefit of having good cognitive abilities occurs when the number of food patches, their values, and the length of the reproductive phase are small. Indeed, natural selection should reduce male cognitive diversity by favoring males with either low or high cognitive ability, when the cost of cognition is high or low, respectively (Figure 4). This arises because the foraging success of males is more variable and, on average, lower when the reproductive phase is shorter. Thus, the cost of cognition has a stronger impact on the reproductive success of the pair. For instance, under short reproductive phases, low cognitive costs should favor males with the greatest cognitive abilities while increasing the cost of cognition should progressively eliminate these males, thereby leading to a reduction in male cognitive diversity (Figure 2Di).

### 3.2 Evolution of cognition under natural and sexual selection

**FIGURE 3:**
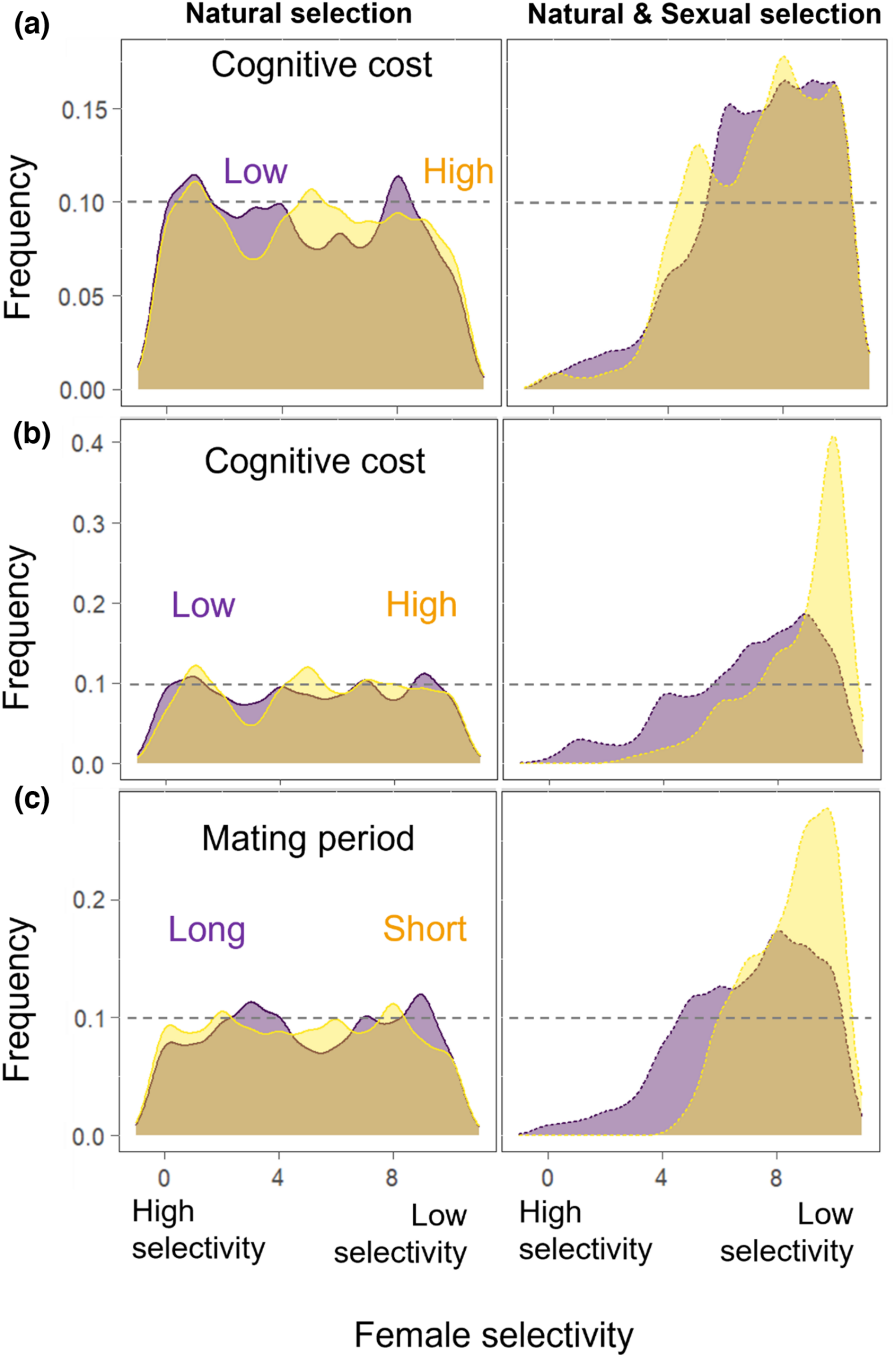
Effect of environmental parameters on the pooled distribution of female selectivity values at the 50th generation from 100 repetitions, under natural selection alone (left panels) or under natural and sexual selection (right panel). Low and high female selectivity values, respectively, mean that the females have a high and low preference for high male cognitive abilities. (a) *Tr* = 200, *Tm* = 200, *R* = 100, *Np* = 85, and *α* is manipulated, (b) *Tr* = 50, *Tm* = 200, *R* = 100, *Np* = 85, and *α* is manipulated, (c) *Tr* = 200, *R* = 10, *Np* = 85, *α* = 1, and *Tm* is manipulated. The purple and yellow colors, respectively, represent low and high values of the manipulated factor. The patterns found in (a) are similar when the number of patches and the number of items per patch are manipulated, and the gray dashed line represents the initial distribution of the trait

We found that intersexual selection always favored males with greater cognitive abilities (Figure 2Aii,Bii,Cii), except when the duration of the reproductive phase was short and the cost of cognition was so large, that males achieving the greatest reproductive success were the ones with the lowest cognitive abilities (Figure 2Dii). Yet, depending on environmental conditions and their effects on the level of selectivity of females and their ability to accurately assess male cognitive trait values, intersexual selection could either have little or no effect, reinforce the effect of natural selection, or act against it (Figure 5; Appendix, Figure A2). Specifically, we found that less accurate females were generally favored, as those females rapidly visited potential mates and, as such, had higher chances to find an acceptable one compared with more accurate ones (Figure 3; Figures A1 and A4). This trend was particularly marked under favorable conditions (i.e., long reproductive period), while greater diversity in female accuracy was maintained in harsher conditions (i.e., short reproductive period, high cognitive costs). Less selective females, logically, were favored when the time available for choosing a mate was reduced. However, increasing the duration of the reproductive phase increased mate opportunity and eroded less diversity in female selectivity (i.e., although the most selective females were eliminated), as such, enhanced the effect of intersexual selection (Figure A3).

**FIGURE 4:**
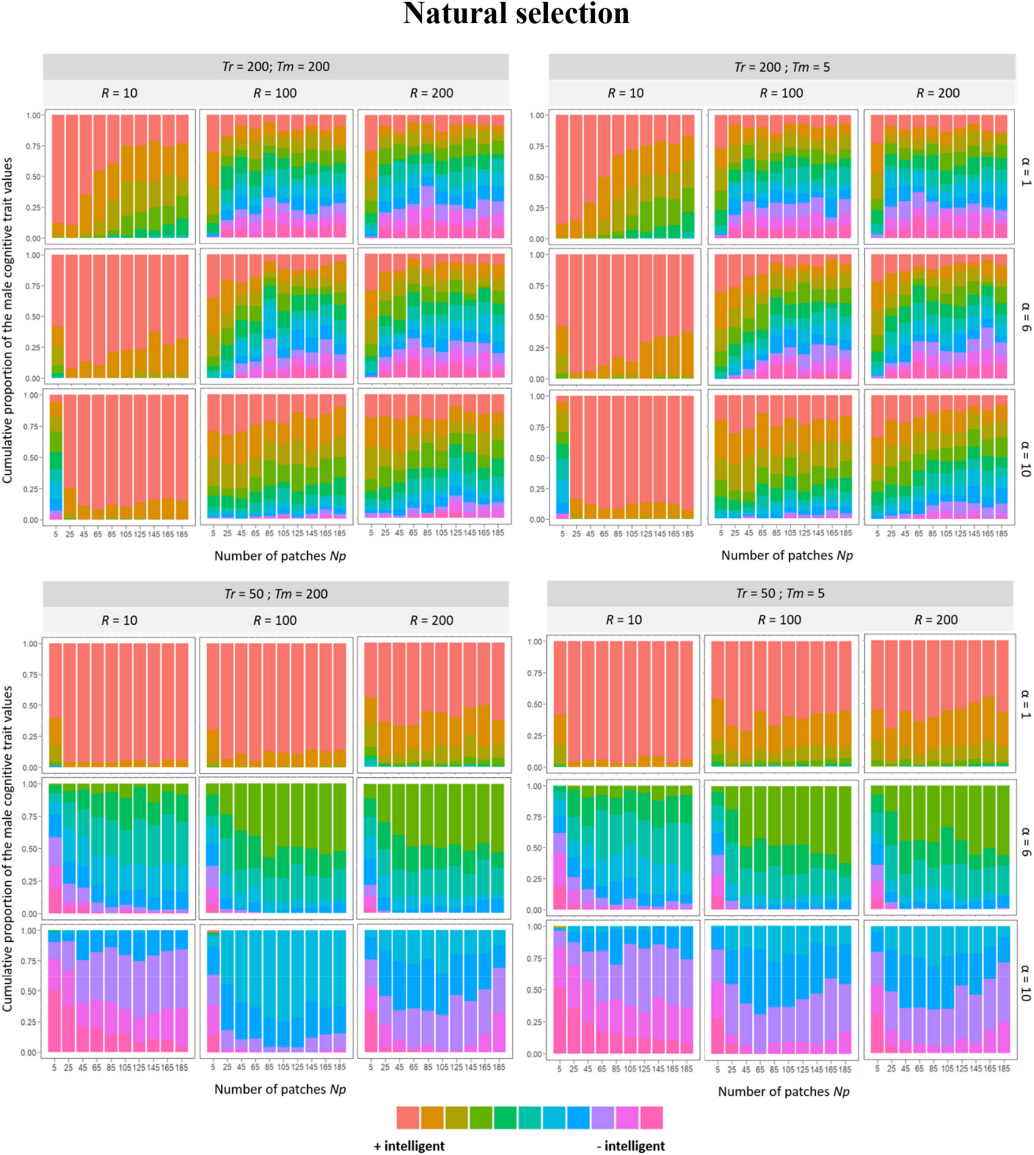
Mean frequency of male cognitive trait values in the 360 environmental conditions in which we made vary the quantity and quality of food patches, the lengths of the reproductive and choosing phases, and the male cognitive cost factor under natural selection from the last 50 generations over 100 repetitions. Each color represents a male trait value from red (i.e., males with a better cognitive ability) to purple (i.e., males with a poorer cognitive ability)

**FIGURE 5:**
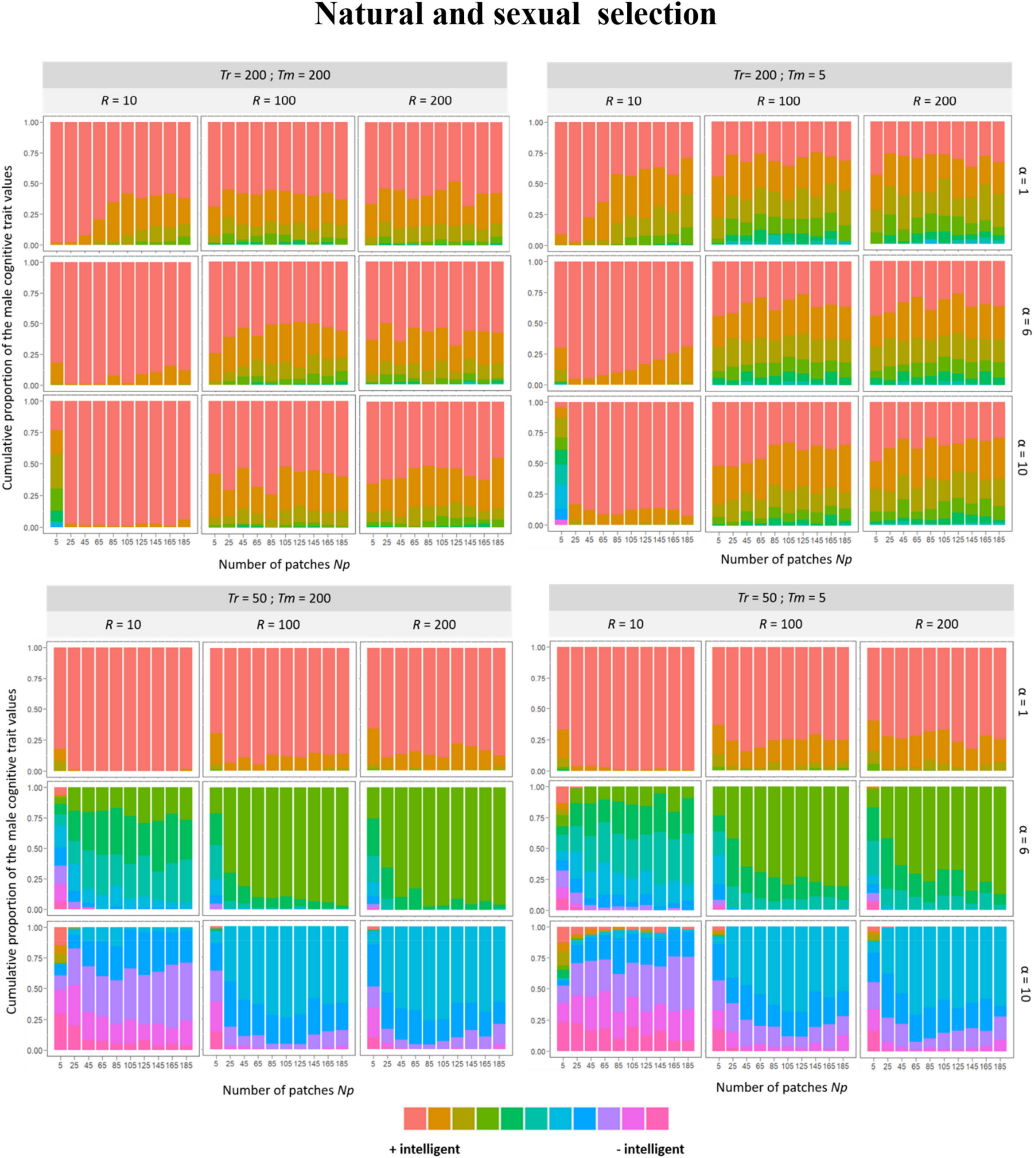
Mean frequency of male cognitive trait values in the 360 environmental conditions in which we made vary the quantity and quality of food patches, the lengths of the reproductive, and choosing phases and the male cognitive cost factor under natural and sexual selection from the last 50 generations over 100 repetitions. Each color represents a male trait value from red (i.e., males with a better cognitive ability) to purple (i.e., males with a poorer cognitive ability)

Thus, when natural selection favored males with high cognitive skills (Figure 2Aii,Bii, Figure A2B), while maintaining enough cognitive diversity for intersexual selection to act on, intersexual selection tended to reinforce the effect of natural selection. In contrast, when natural selection eliminated almost all the cognitive diversity by favoring males with higher cognitive abilities, the intersexual selection had inevitably almost no effect (Figure A2A). This was the case notably when the food patches were poor and limited, the length of the reproductive phase was short, and the cost of cognition was weak (Figure 5). Inversely, when natural selection alone favored males with poor cognitive abilities, intersexual selection tended to eliminate, at least partially, these males who were progressively replaced by individuals with higher cognitive abilities (Figure A2C). In fact, more accurate and selective females should select males with good abilities while less accurate and less selective females should select males with poor abilities. Such a scenario, in which intersexual selection should act against natural selection and maintain cognitive diversity, occurs in the harshest environments (i.e., poor patches, short mate choice, and reproductive phases) when the cost of male cognition is large (Figure 5, Figures A3 and A4).

## 4 DISCUSSION

Our results demonstrate that the relative importance of natural and sexual selection on male cognition and female assessment strategies depends on environmental conditions. Thus, our study highlights the importance of considering the interaction between both selective forces, as ecological conditions experienced by individuals act on the variance in male cognitive traits and hence on the potential for sexual selection. Specifically, under natural selection alone, we found that the evolution of male cognition was mainly driven by the intensity of resource competition and the cost of male cognition. When food resources were limited (i.e., under strong competition), natural selection tended to favor males with high cognitive abilities that had privileged access to them. By contrast, when resources were abundant (i.e., under weak competition), males could easily find food and, regardless of their cognitive skills, mostly achieved the maximal reproductive score. Thus, under such conditions, natural selection had a weak effect and maintained most of the initial cognitive diversity. These predictions agree with several theoretical and experimental studies demonstrating that social competition is an important driver of the evolution of cognition (e.g., Morand‐Ferron et al., 2015; Pravosudov & Roth, 2013; Szabo et al., 2020). For instance, Pravosudov and Roth (2013) reported that food‐caching black‐capped chickadees (*Poecile atricapillus*) facing severe winter conditions are more accurate in a spatial memory task and faster in habituation and problem‐solving tasks, compared with individuals facing milder winter conditions. However, as cognition is costly, in terms of production and maintenance of neuronal and physiological processes involved in cognitive mechanisms (Dukas, 1999; Jaumann et al., 2013; Laughlin et al., 1998), high cognitive abilities should be favored only when the benefits, notably in terms of foraging success, exceed the costs (Niemelä et al., 2013). In support, we found that natural selection decreased male cognitive diversity within populations when the reproductive phase was short, by, respectively, promoting high, intermediate, and low cognitive abilities, when the cost of cognition was low, intermediate, and high. These predictions are also aligned with numerous studies showing that, when time or energy available is restricted, cognitive investments can trade off with other fitness‐related traits, such as development time (Christiansen et al., 2016; Snell‐Rood et al., 2011), lifespan (Burger et al., 2008), competitive abilities (Mery & Kawecki, 2003) or immune response (Alghamdi et al., 2008; Gegear et al., 2006; Iqbal & Mueller, 2007) and thus limit their adaptive value. For instance, honeybees suffer from significant cognitive impairment under energetic stress (Jaumann et al., 2013) suggesting that trade‐offs with energy use can prevent good cognitive abilities from evolving in populations (Dunlap & Stephens, 2016).

The expected patterns of evolution under natural selection changed under most conditions when including the effect of intersexual selection. Indeed, we found that intersexual selection had no or very little effect only when natural selection eroded most of the variance in male cognitive abilities, which happened when resource competition was strong and the cost of cognition was low. In all other conditions, intersexual selection through female mate choice either reinforced or acted against the effect of natural selection. Specifically, intersexual selection reinforced the effect of natural selection when the male cognitive ability was a reliable indicator of the reproductive success of the pair (Boogert et al., 2011). This happened when the reproductive phase was long because the variance in male foraging success was then very large, resulting in a strong positive correlation between male cognitive trait value and breeding success. Obviously, a long choosing phase increased the females' likelihood of finding an acceptable mate. Thus, the effect of intersexual selection was even stronger when the most selective females were maintained within the population. This, however, occurred very rarely as we did not allow unmated females to decrease their acceptance threshold at the end of the reproductive phase or to have a last mating chance (e.g., Janetos, 1980). Also, because we have assumed that males can have only one mating partner and the sex ratio is balanced, we have imposed a very strong competition among females. For these reasons, we found that the least accurate, but most rapid females were favored in most conditions, and that intersexual selection had a weak effect on female assessment strategy. The strength of intersexual selection, however, is likely stronger under natural populations, than that we predict. Further studies would then be required to better understand the effect of sexual selection on male cognition and female assessment strategy in relation to social mating system conditions. Interestingly, when natural selection alone favored males with low cognitive abilities, intersexual selection acted against natural selection by favoring the extreme male cognitive trait values and promoting the maintenance of diversity in female traits determining assessment strategy (i.e., selectivity and accuracy). Under such conditions, population was made up of males with high cognitive abilities that were very efficient at finding food but suffered a large cost of cognition, and of males with low cognitive abilities that obtained food at a lower rate but suffered no cost. Thus, selective and accurate females that preferred males with greater cognitive traits, achieved similar reproductive success to less selective and less accurate ones. As such, our results support the idea that cognitive diversity can be maintained by intersexual selection through female mate choice (fish: Álvarez‐Quintero et al., 2021; birds: Barou‐Dagues et al., 2020; Barou‐Dagues & Dubois, 2022; humans: Escorial & Martín‐Buro, 2012; Plomin & Deary, 2015; Śmieja & Stolarski, 2018). Although more evidence is needed to determine which ecological conditions contribute to maintaining differences in female preference for male abilities, our predictions are nevertheless consistent with the fact that no study so far has demonstrated an unanimous female preference for male cognitive abilities.

More generally, our study supports the idea that intersexual selection is an important driver of the evolution of cognition and highlights the need of building a conceptual framework to target relevant traits. Indeed, since correlations between foraging abilities, noncognitive traits, and individual performances on different cognitive tasks may vary among species (Burkart et al., 2017; Poirier et al., 2020), sexual selection should favor different sets of skills depending on the female needs and thereby on the ecological and social mating system conditions. For instance, the qualitative predictions of our model may apply to any (cognitive or noncognitive) trait that is potentially costly and directly affects the speed at which males can acquire food items and, as such, their breeding success. However, our predictions would likely change by introducing variations in the sex ratio or mating system. Under unbalanced sex ratio, for instance, or under a scenario where population size would increase across generations (e.g., low predation threat), we might expect stronger competition among females to access a partner and thus stronger intersexual selection on male and female traits. Conversely, under promiscuous or polyandrous mating systems, male mating opportunities would be increased leading to a reduction in the strength of intersexual selection. Further experimental and meta‐analytic studies are then required to better understand how natural selection interplays with sexual selection under different ecological environments and across mating systems when females may obtain both direct and indirect fitness benefits. Experiments designed to assess the heritability of cognitive trait measures (e.g., Smith et al., 2015) will also be useful to make more realistic predictions concerning the relative importance of both selective forces. In our simulation model, indeed, we have assumed that cognitive traits and female preferences are fully heritable, thereby increasing the rate at which natural and sexual selections operate. Thus, investigating how genetic (Croston et al., 2015; Lagnely, Adams, et al., 2020) and nongenetic (i.e., maternal effect: Basatemur et al., 2012; Munch et al., 2018; social context: Lagnely, Adams, et al., 2020; Langley et al., 2018a, 2018b) factors affect the heritability of cognitive traits and hence their rate of evolution would allow to better understand to what extent differences in cognition reflect adaptations to ecological conditions.

## AUTHOR CONTRIBUTIONS

**Marie Barou‐Dagues:** Conceptualization (equal); data curation (lead); formal analysis (lead); funding acquisition (supporting); investigation (equal); methodology (lead); project administration (lead); resources (lead); software (lead); supervision (equal); validation (lead); visualization (lead); writing – original draft (equal); writing – review and editing (equal). **Frédérique Dubois:** Conceptualization (equal); data curation (supporting); formal analysis (supporting); funding acquisition (lead); investigation (equal); methodology (supporting); project administration (supporting); resources (supporting); supervision (equal); validation (supporting); visualization (supporting); writing – original draft (equal); writing – review and editing (equal).

## CONFLICT OF INTEREST

The authors declare that they have no conflict of interest.

## Supporting information


Appendix S1

## Data Availability

The code of the simulation is accessible in Supplementary material B. The data extracted from the model are openly available on Dryad (https://doi.org/10.5061/dryad.bcc2fqzfx).
