# Supplementary material for: Exploring the interplay between natural and intersexual selection on the evolution of a cognitive trait
Source: Ecol Evol. 2022 Jul 4;12(7):e9066. doi: 10.1002/ece3.9066 (PMC9251863; doi:10.1002/ece3.9066)
Supplement: Supplementary file 1 — Appendix S1 [file ECE3-12-e9066-s001.zip › ECE3_9066_sm_0001-Supinfo.docx]

**Appendix**


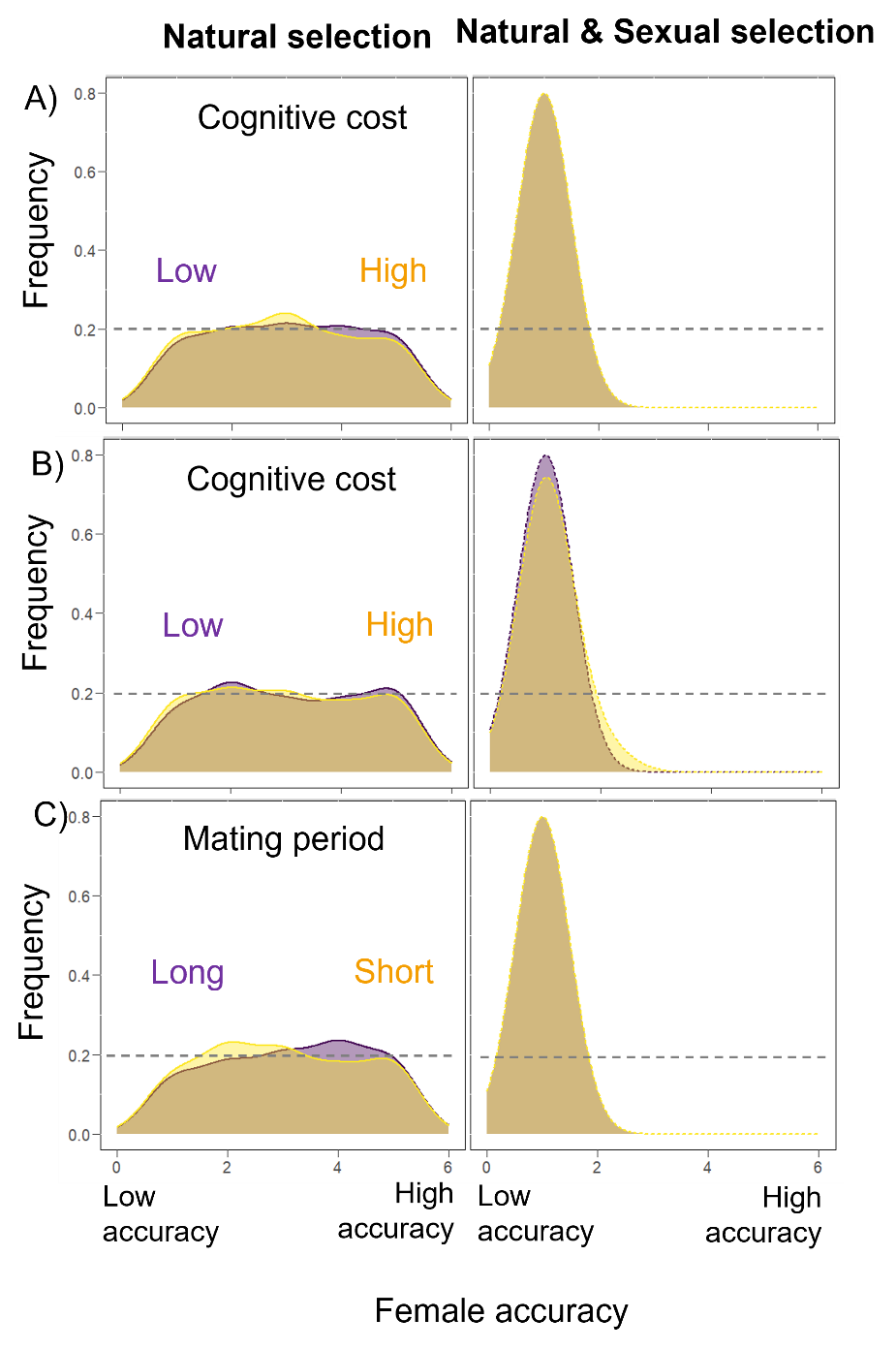


Figure A1. Effect of environmental parameters on the distribution of female accuracy values at the 50th generation under natural selection alone (left panels) or under natural and sexual selection (right panels). Low and high female accuracy values respectively mean that females make no and large assessment errors. A) *Tr* = 200, *Tm* = 200, *R = 100, Np* = 85 and *α* is manipulated, B) *Tr* = 50, *Tm* = 200, *R = 100, Np* = 85 and *α* is manipulated, C) *Tr* = 200, *R* = 10, *Np* = 85, *α* = 1 and *Tm* is manipulated. The purple and yellow colors respectively represent low and high values of the manipulated factor. The patterns found in A) are similar when the number of patches and the number of items per patch are manipulated and the grey dashed lines represent the initial distribution of the trait.


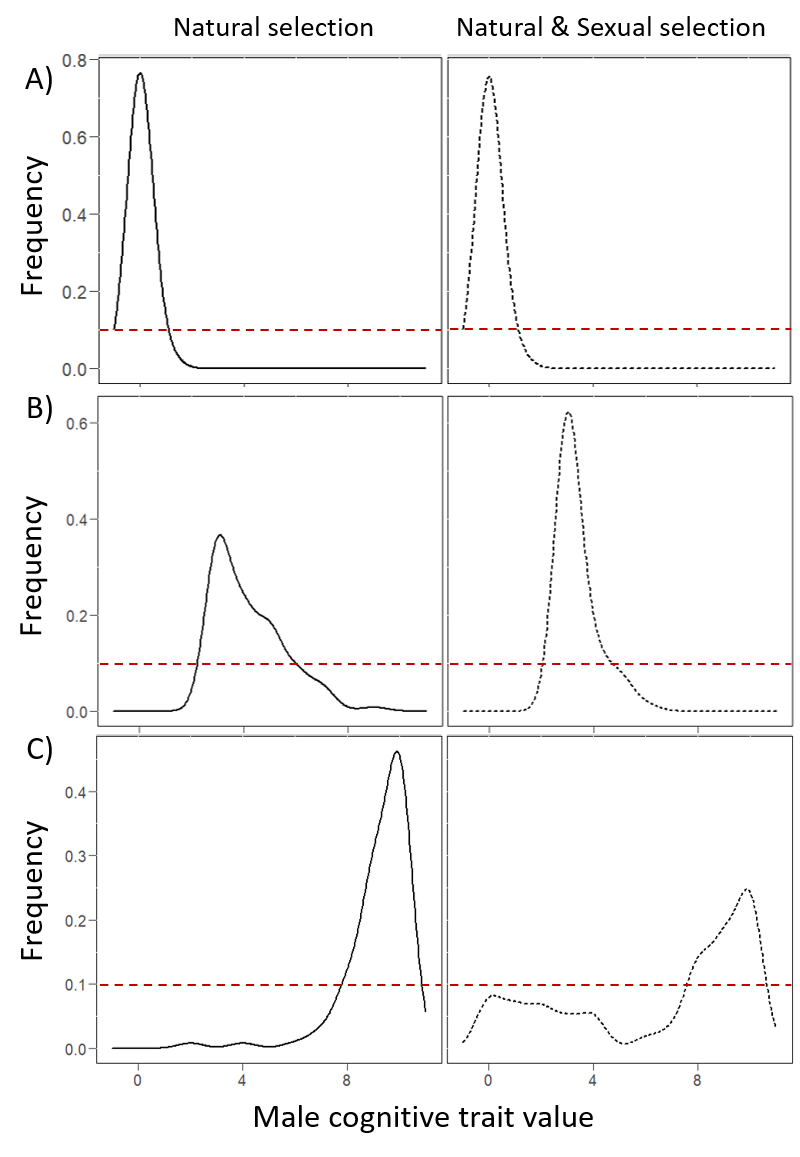


Figure A2. Pooled distribution of male cognitive trait values at the 50th generation from 100 repetitions, under natural selection alone (solid lines) or under natural and sexual selection (dashed lines). Plot A: *Tr* = 50, *Tm* = 200, *Np* = 185, *R* = 100 and *α* = 1, Plot B: *Tr* = 50, *Tm* = 5, *Np* = 105, *R* = 100 and *α* = 6, Plot C: *Tr* = 50, *Tm* = 5, *Np* = 5, *R* = 10 and *α* = 10. Low and high male cognitive trait values respectively mean that the males have high and low cognitive abilities, and the red dashed line represents the initial distribution of the trait.


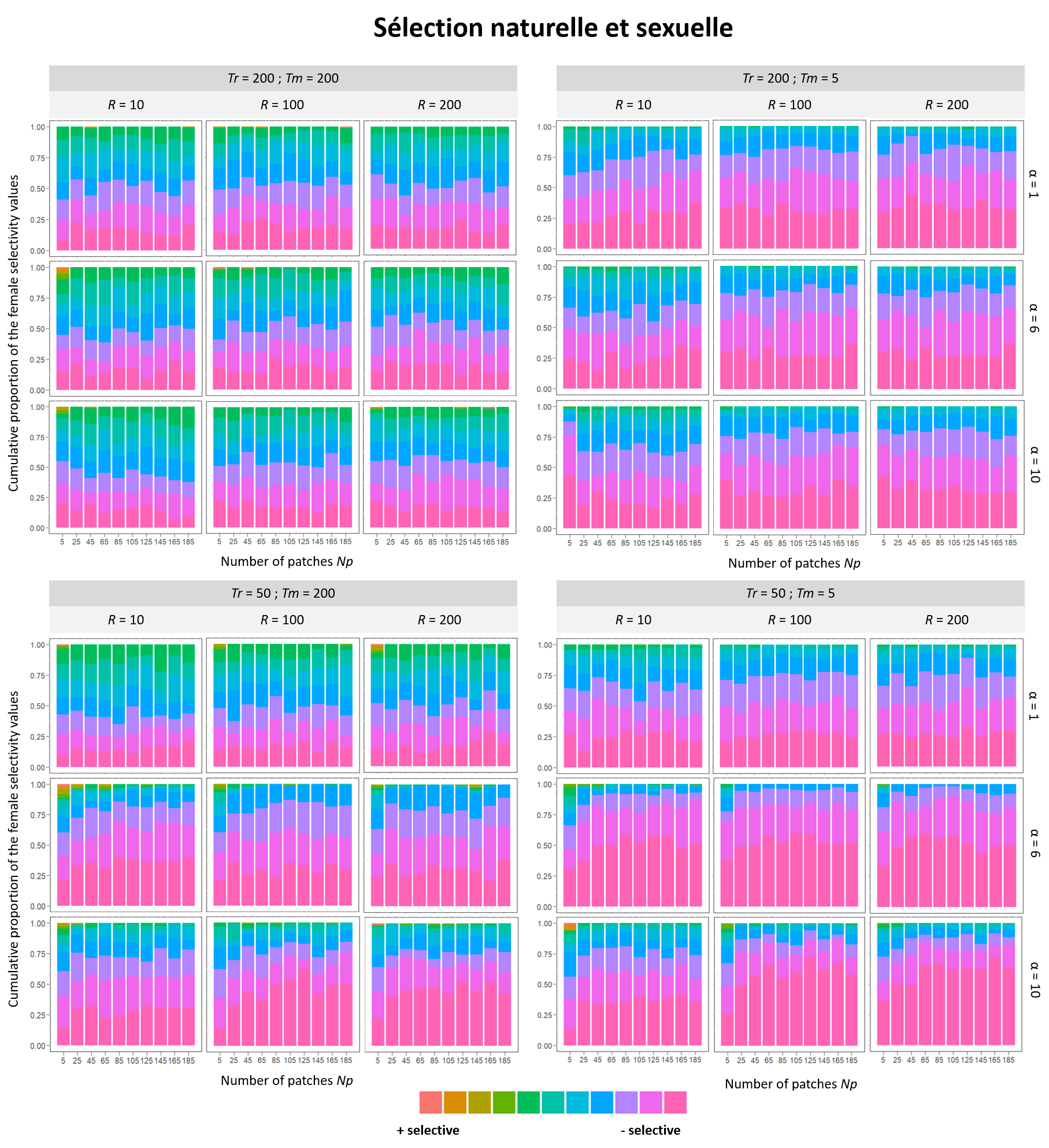


Figure A3. Mean frequency of female selectivity values in the 360 environmental conditions in which we made vary the quantity and quality of food patches, the lengths of the reproductive and choosing phases and the cost of male cognition under natural and sexual selection from the last 50 generations over 100 repetitions. Each color represents a female trait value from red (i.e. females with high selectivity threshold) to purple (i.e. females with low selectivity threshold).


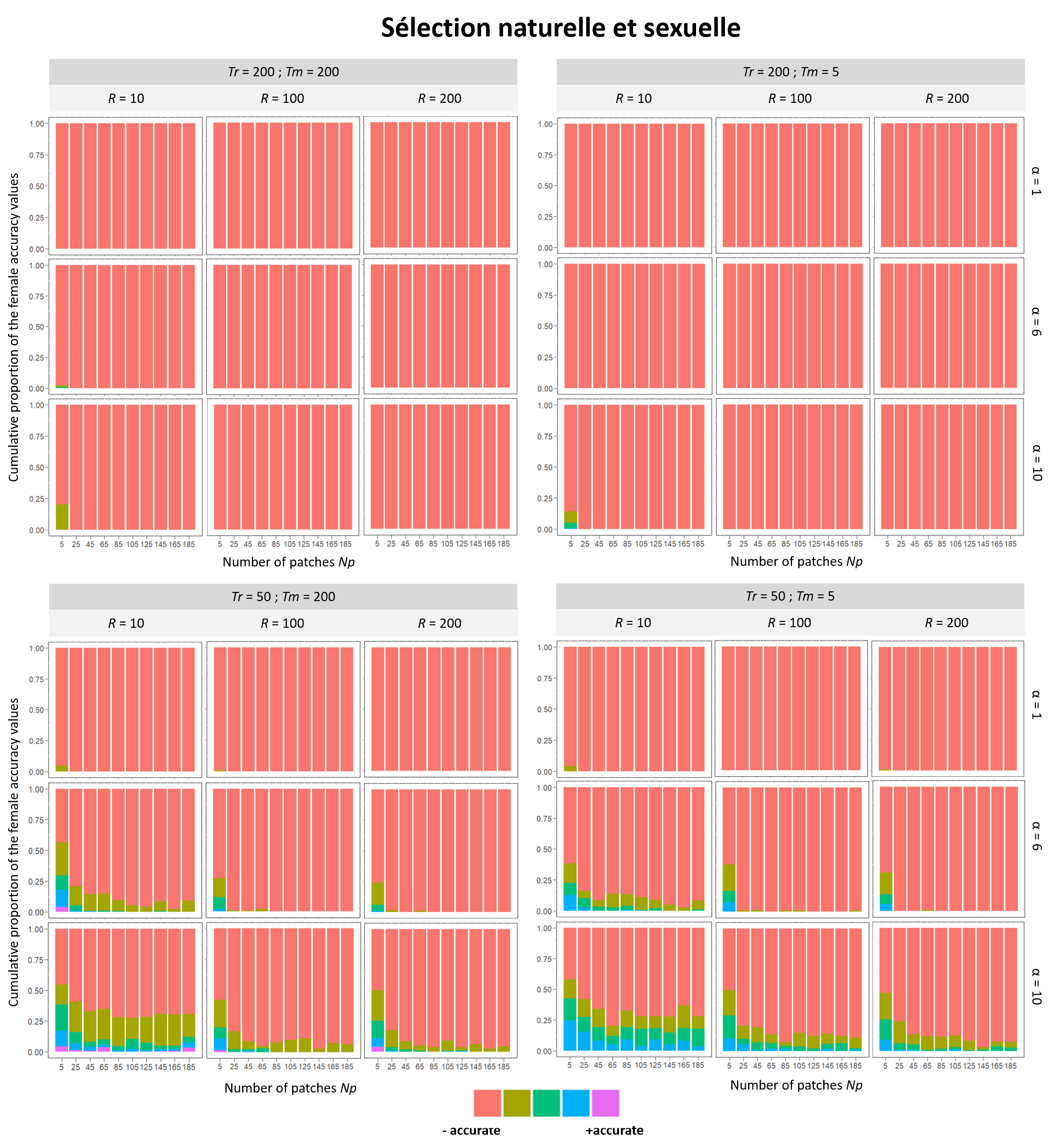


Figure A4. Mean frequency of female accuracy values in the 360 environmental conditions in which we made vary the quantity and quality of food patches, the lengths of the reproductive and choosing phases and the cost of male cognition under natural and sexual selection from the last 50 generations over 100 repetitions. Each color represents a female trait value from red (i.e. females with low accuracy ability) to purple (i.e. females with high accuracy ability).
