# Supplementary material for: Exploring the interplay between natural and intersexual selection on the evolution of a cognitive trait
Source: Ecol Evol. 2022 Jul 4;12(7):e9066. doi: 10.1002/ece3.9066 (PMC9251863; doi:10.1002/ece3.9066)
Supplement: Supplementary file 1 — Appendix S1 [file ECE3-12-e9066-s001.zip › ECE3_9066_Supplmementary materials.docx]

**Exploring the interplay between natural and intersexual selection on the evolution of a cognitive trait**

**Supplementary materials**

ODD protocol

The model description follows the ODD (Overview, Design concepts, Details) protocol for describing individual- and agent-based models (Grimm et al., 2006), as updated by Grimm et al. (2020).

A. Description of the model

1. Overview
   1. *Purpose and patterns*

The general purpose of the model is to predict the relative strength and direction of natural and intersexual selection on the evolution of a male cognitive trait that contributes to females’ reproductive success. The model aims at identifying whether and when intersexual selection reinforces natural selection, acts against it or has no effect on the evolution of cognition. Specifically, it aims at comparing the distribution of male abilities to resolve a problem, in absence and presence of female mate preference for this male cognitive trait, under different sets of environmental scenarios. We evaluate our model by its ability to reproduce three patterns: 1) a positive relationship between the foraging success of the male and the reproductive success of the pair, 2) female preference for males with greater cognitive abilities and 3) heritability of male and female cognitive abilities.

Pattern 1: positive relationship between foraging success and reproductive success. This pattern reflects the father’s provisioning rate during nestling development. In monogamous species with biparental care, both parents invest in parental care and nestling growth and survival are often directly related to the time and energy the father invests in provisioning the offspring with food (Wetzel, 2017).

Pattern 2: female preference for male cognitive abilities. In several mammal and bird species, females that can choose among males that differ in terms of cognitive abilities, demonstrate a mate preference for males with higher cognitive abilities (Chen et al., 2019). In this model, we simulate the effects of both natural and intersexual selection by allowing females to choose a mate according to its cognitive trait value.

Pattern 3: heritability of male and female cognitive abilities. In human and several nonhuman animal species, cognitive traits are, to some extent, heritable (e.g. Croston et al., 2015; Hopkins et al., 2014). For simplicity, we set a perfect trait heritability for male and female cognitive traits.

- 1. *Entities, state variables and scales*

This model includes two different types of agent populations (‘male’ or ‘female’), each always containing 100 agents. Within the male population instance, each *male* *agent* is characterized by a static integer ID, a dynamic spatial position on the grid [x, y], a static cognitive trait value (*I_i_*) ranging from 0 to 10 (i.e. number of time steps required to solve a problem and gain access to the food), a static cognitive cost ($\propto\left[ 10-I_{i} \right])$ ranging from 0 to 100, a dynamic integer foraging success (i.e. number of food items eaten during the foraging period minus the cognitive cost), a dynamic boolean foraging status (i.e. over a food patch: true/false), a dynamic boolean mating status (i.e. available: true/false) and a dynamic integer reproductive success ranging from 0 to 11 that represents the number of offspring produced. Within the female population instance, each *female agent* is characterized by a static integer ID, a dynamic spatial position on the grid [x, y], a static accuracy variable *A_j_* ranging from 0 to 5 (i.e. number of time steps required to assess males’ cognitive trait value), a static selectivity variable *S_j_* ranging from 0 to 10 (i.e. minimum acceptance value during mate choice), a dynamic integer foraging success that they acquire from their partner after mating, a dynamic boolean mating search status (i.e. over a male territory: true/false), a dynamic boolean mating status (i.e. found mate: true/false), a static mating strategy (i.e. under natural and sexual selection: mate when the estimate of the male cognitive trait is lower or equalto *S_j_*.; under natural selection alone: mate with the first male encountered) and a dynamic integer reproductive success ranging from 0 to 11.

The model also includes a *temporal scale* that consists of 1000 consecutive generations, each of them characterized by a reproductive season composed by a choosing phase (*Tm* time steps) during which females visit male territories one after another and choose a mate, and a reproductive phase (*Tr* time steps) during which males move one step at a time from one territory to another, search for food and provision their young.

Two spatial environment instances are also included: the foraging and the mating environment instances, both characterized by a two-dimensional grid containing 14x14 territories. Each environment instance is characterized by its type (i.e. food patch territories or male territories) and a number of territories (i.e. number of food patches *nP* or number of males). Within the *foraging environment* instance, each food patch is characterized by a static integer ID, a static spatial position [x, y] on the grid, a dynamic integer richness (i.e. number of food items in the patch) and a dynamic boolean value (presence of food: true/false). The mating *spatial environment* corresponds to the males’ territories and are characterized by the males’ state variables at the end of the foraging period.

1. Process overview and scheduling

*Processes*: The simulation is developed to cover the evolution of cognition over 1000 consecutive generations. It is structured along six processes: the foraging environment (creation, characterization and set position of the food territories), the foraging time loop (males search for food patches), the mating environment (creation, characterization and set position of the mating territories), the mating time loop (females search and mate with partners), the reproductive success of breeding pairs (selection of breeding pairs and reproductive success assignment) and the creation of the next generation (production of offspring and transmission of traits from parents to offspring, replacing the parents by offspring for the next generation).

The foraging environment is created and characterized before the foraging time loop. The foraging environment and the male agents update their state variables at each foraging time step. The mating environment is created and characterized before the mating time loop. The mating environment and the female agent update their state variables at each mating time step. The production of the next generation is updated at the end of the mating loop time.

We repeated the simulation over 100 repetitions to control for stochastic effects. These 100 repetitions were run under 360 different constant foraging environments. We made vary: the male cognitive cost factor $\propto$ (1, 6 or 10), the length of the foraging and mating phases, *Tf* (200 or 50) and *Tm* (200 or 5), and the richness of the patches *rP* (10, 100 or 200) and the number of food patches (through incrementing *nP* by 20 after each 100 repetitions). We ran two different models, one predicting the evolution of cognition under natural selection alone and the other predicting the evolution of cognition under natural and intersexual selection. Both models are identical except for the female mating strategy. Under natural selection alone, all females mate with the first male encountered during the foraging period while under natural and sexual selection, females mate with the first encountered male whose estimated cognitive trait value is equal or lower than their selectivity threshold.

*Schedule*: Before the beginning of the simulation, an initial population composed by 100 males and 100 females is characterized. The foraging environment is scheduled first because subsequent males’ foraging behaviour depends on environmental conditions. The foraging time loop occurs before the mating environment and mating time loop because we used the males’ state variables to characterize the mating environment so females can move from one male territory to another. Biologically, we explain in the manuscript that females first choose their mate according to their cognitive abilities and then males search for food, provide their young with food and, as such, determine the reproductive success of the pair. In the simulation model, males and females’ behaviour are independent in the foraging and mating time loops. It means that running either the foraging or the mating time loop first does not affect the data per se. Because the state variables and agents change between the foraging and mating environments, and because we used the male state variables to characterize the mating environment, for simplicity, we chose to run first the foraging loop and then the mating loop.

1. Foraging environment (creation of the grid, characterization of the food patches, set of food patches’ initial position)

2. Foraging time loop

2.1 Update each male position on the grid and compare its new coordinate with the location of the food patches.

2.2 If a male is over a food patch, he executes its “feeding behaviour”, which includes:

2.2.1 comparing the number of time steps spent by the male over this patch with his cognitive ability to find food

2.2.2 if both are equal, incrementing by 1 the male foraging success and removing 1 item from the patch richness, otherwise the male stays at the patch for another time step.

3. Mating environment (creation, characterization and set of initial position of the male territories)

4. Mating time loop

4.1 Update each female position by randomly moving to one male territory to another and check whether each female encounters an available male

4.2 If yes, the female executes her “mating behaviour” which includes:

4.2.1 comparing the number of time steps spent by the female in front of the male with her degree of accuracy

4.2.2 if both are equal, the female estimates the male cognitive trait value with an error inversely proportional to her degree of accuracy

4.2.3 if her estimate is inferior to her selectivity threshold, the female mates with the male, acquires his foraging success, and both partners become no longer available.

5. Selection of breeding pairs and attribution of reproductive success scores: zero to individuals whose foraging success is equal or less than zero, and a score of 1 offspring produced for every 10 units of foraging success for the other individuals with a maximum reproductive success of 11.

6. Creation of the next generation

6.1 Production of the offspring

6.2 Reinitialization of the agent’s characteristics for the next generation by using the selected offspring’s traits values (i.e. male cognitive trait, female selectivity and female accuracy).

1. Design concepts
   - 1. *Basic principles*

At the system level, the model addresses a general question about the respective role of natural and sexual selection on the evolution of cognition. Specifically, the evolution of cognition under natural selection has been extensively studied (e.g. Morand-Ferron & Quinn, 2015) whereas the importance of sexual selection is still debated. In natural populations, the respective role of natural and intersexual selection on the evolution of cognition has not been investigated yet. This model is the first to our knowledge to predict the relative effect of both forces on the evolution of cognitive abilities at the intraspecific level.

In its foraging loop submodel, this model poses a common question about the adaptive value of animal problem-solving abilities under natural selection: what and how environmental factors affect the ability to resolve a problem at the intraspecific level (Raine & Chittka, 2008)? In addition, in mating loop submodel, the female behaviour is based on extensive literature about how females assess male sexual traits and how female differences in selectivity and accuracy affect their mate preferences (Rosenthal, 2017). Based on recent studies revealing the presence of mate choice for cognitive traits (Boogert et al, 2011), this model asks whether female preference for males with high cognitive abilities changes the evolutive pattern observed when females do not express this mate preference.

- - 1. *Emergence*

The distribution of the male cognitive abilities and female selectivity and accuracy trait values emerge as unmated individuals as well as mated individuals with lower foraging success produce less offspring and are progressively eliminated from the population. When the evolution of cognition is predicted under natural selection alone, the model forces the females to mate with the first male encountered and causes the absence of evolutive patterns for female traits. These imposed results allow us to confirm that no sexual selection is acting on the male cognitive trait.

- - 1. *Adaptation*

Under natural selection alone, the model assumes that natural selection eliminates individuals with a negative or null foraging success at the end of the foraging period and individuals that do not find a mate at the end of the mating period. For breeding pairs, it allows individuals to produce 1 offspring for every 10 units of foraging success. In this case, higher male cognitive abilities can evolve only if they are associated with increased foraging success in the given ecological conditions. Under natural and sexual selection, the model assumes in addition that females prefer males with high cognitive abilities so that these males have better chances compared to the others to mate before the end of the mating period.

- - 1. *Objectives*

The objective measure used by the male agent to be able to produce offspring is the foraging success. The foraging success is initialized to 0 at the beginning of each foraging period and is incremented by 1 each time it gets a food item. At the end of the foraging period, the male cognitive cost ($-\propto\left[ 10-{Cogn}_{i} \right]$) is deducted from the number of food items consumed by the male. Because the foraging success is often associated with offspring survival (Wetzel, 2017), this objective measure allows each mated partner to provide with food for 1 offspring for every 10 units of foraging success with a maximum of 11 offspring.

- - 1. *Learning*

Learning is not implemented.

- - 1. *Prediction*

Individuals do not change their behaviour or decision rules over time because of their experience and they cannot predict future conditions.

- - 1. *Sensing*

Males are assumed to perfectly detect the presence/absence of food in a patch even if they do not have yet resolved the problem accessing it. Females are assumed to know the location of all male territories and to detect the presence of a potential mate. Females do not perfectly assess the male cognitive trait but differ in their ability to accurately assess this trait (i.e. accuracy). Less accurate females are faster whereas more accurate females are slower to estimate the male cognitive trait value.

- - 1. *Interaction*

During the foraging period, males interact indirectly with each other by competing for limited food items per patch during a limited foraging period. Males vary in their ability to gain access to the food (male with higher cognitive abilities are faster to resolve the problem and access the food), can consume only one food item at each time step and a food patch can be exploited simultaneously by several males. During the mating period, females interact indirectly with each other because as soon as a female mates with a male, he is no longer available to mate for other females. In addition, females interact with males because they visit one male territory after another, assess each male cognitive trait value, and when they accept to mate with a male, they produce a number of offspring proportional to the foraging success of their mating partner. Thus, females’ reproductive success is determined by the foraging success of their partner while males’ reproductive success depends on whether or not they are chosen by a female to reproduce.

- - 1. *Stochasticity*

At the beginning of the foraging phase, each male is randomly assigned to a territory and can move from one territory to another using a random walk. Food patches are randomly assigned to available positions on the grid and once a food patch is depleted, it is immediately replaced by another one, whose location is chosen randomly among all unoccupied and empty territories. At the beginning of the mating phase, males and females in turn are randomly assigned to a new unique location on the grid and females can randomly move from one male territory to another. For the first generation, male and female traits are randomly drawn from discrete uniform distributions. Subsequently, the male and female offspring inherit, respectively, their father’s and mother’s trait values. The 100 sons and 100 daughters are randomly selected among all offspring produced at the end of each reproductive season to keep the population size constant. To account for stochastic effects, the same simulation was run 100 times. Also, by completely renewing the population at each generation, we imposed a very strong selective pressure and increase the bottleneck effect. For this reason, we only studied the evolution of cognitive traits after 50 generations (the predicted patterns remain unchanged if we look at the variance among populations between 950 and 1000 generations).

- - 1. *Collectives*

The model includes no collectives.

- - 1. *Observation*

For model testing, MB-D debugged the model and used the print function to find and fix all potential mistakes that could have occurred at each step of the simulation. MB-D also made numerous tests to make sure that the code stopped running after setting some variables with wrong values or when no females had found a mate. As mentioned in the *Emergence* section*,* the model forces the females to mate with the first male encountered under natural selection alone. In that case, therefore, there is no evolutive pattern for female traits and the obtained results thus allowed us to insure that no sexual selection was acting on the male cognitive trait.

In order to predict the relative importance of natural and sexual selection on the evolution of cognition under different scenarios, we extracted 3 output data to look at distributions of male cognitive ability and female selectivity and accuracy values across all agents (100 males and 100 males), for each generation (1000 generations in total), for each repetition (100 repetitions in total), for each scenario (360 different environments) and for both models with and without intersexual selection. Each scenario is a combination of five parameters we manipulated before running the simulation (see *Initialization*). We also extracted another output data to look at the mean frequency of each trait values for the last 50 generations (i.e. from 950 to 1000) for each repetition and for each scenario.

1. Details
   1. *Initialization*

Before the simulation, each male is assigned an ID, a spatial position, a cognitive ability, a foraging success, a foraging status, a mating status, a fitness category and a reproductive success and each female is assigned an ID, a spatial position, a selectivity value, an accuracy value, a foraging success, a mating search status, a mating status, a fitness category, a reproductive success. These state variables are initialized before the simulation to create an initial population. The initialization method of the foraging and the mating environment (see *Submodels*) is the same from one scenario to another. The value of the environment state variables is kept constant for a given scenario (across 1000 generations and 100 repetitions) but differ across the different scenarios. Several environmental parameter values are changed before running the model. Specifically, we specify the foraging time step number, the mating time steps number, the number of items per food patch and the male cognitive cost before starting the simulation. Also, the simulation is iterated over 10 different values of the number of patches given rise to 360 different environmental scenarios. We chose these five parameters because they are known to affect individual cognition and the value of each parameter was chosen to cover the maximal range of environmental conditions (i.e. from favourable to very harsh environments).

- 1. *Input data*

The environment is kept constant, so the model has no input data.

- 1. *Submodels*

*Foraging environment*: The foraging environment instance is a list created and populated by *Np* number of food patches, each characterized by an ID (index + 1) and each containing the same *R* initial number of items. The spatial position on the grid is then set for each food patch such as each unique coordinate [x, y] is randomly selected among all available territories from the 14 x 14 two-dimensional grid.

*Foraging time loop:* At the beginning of the foraging time loop, the agent population instance contains 100 males, each characterized by an ID, a randomly selected coordinate selected among all available territories from the 14 x 14 two-dimensional grid, a random selected cognitive ability value, a foraging and reproductive success set to zero, a foraging status initialized at “false” and a mating status initialized at “available”. At the beginning of each time step, each male that is not on a patch that contains food updates its position [x, y] by randomly moving to one of the territories located in the 4 cardinal directions. Then, each male checks whether its coordinate is equal to the coordinate of one of the food patches in the foraging environment instance. If this is the case, the male changes its foraging status by becoming ‘true’ and the ID of the food patch is identified. Then, each male with a foraging status ‘true’ checks whether the patch contains a positive number of food items. If yes, the male compares the number of time steps spent on this patch with its cognitive ability. If it is inferior to its cognitive ability, he stays at this patch for another time step, otherwise he consumes one food item (and so its foraging success is incremated by 1) and the food patch loose one food item (the richness of the patch is decremented by 1). If the patch is empty, the male’s foraging status turns to ‘false’, a new food patch, whose position is randomly chosen among all unoccupied and empty territories, is filled with *R* food items.

*Mating environment:* The mating environment instance is a list created and populated by the 100 males that previously behaved in the foraging time loop. Each male territory is characterized by the male’s state variables at the end of the foraging time loop. The cognitive cost of each male is subtracted from the total number of items consumed by the male at the end of the foraging period to obtain the final foraging success of each male. A new spatial position on the grid is then set for each male in a way that a unique coordinate [x, y] is randomly selected among all available male territories from the 14 x 14 two-dimensional grid.

*Mating time loop:* At the beginning of the mating time loop, the agent population instance contains 100 females, each characterized by an ID, a randomly coordinate selected among all available territories from the 14 x 14 two-dimensional grid, a random selected selectivity value, a random selected accuracy value, a null foraging and reproductive success, a mating search status initialized to ‘false’ and a mating status initialized to ‘false’. At the beginning of each time step, each unmated female that is not on a male territory updates its position [x, y] by moving to a randomly selected male territory. Then, each female checks whether her coordinate is equal to the coordinate of an available male. If this is the case, the female’s mating status is changed to ‘true’ and the ID of the male territory is identified.

*#With intersexual selection*, the female checks whether the time spent on the male’s territory is inferior to her accuracy ability (i.e. number of time steps the female need to estimate the male trait). If yes, she stays another time step on the territory, otherwise she estimates his cognitive ability *E_i_* with: *E_i_* = *I_i_* ± *(*5 *– A_j_).* She then checks weather her estimate meets her selectivity threshold. If so, her mating status changes to ‘true’, the male mating status changes to ‘false’ and the female acquires the foraging success of her mating partner. If her estimate does not meet her selectivity threshold, her mating search status turns to ‘false’.

*#Without intersexual selection*, the female’s mating status changes to ‘true”, the male mating status becomes ‘false’ and the female acquire the foraging success of her mating partner.

*Reproductive success of breeding pairs:* At the end of the mating period, all mated individuals are selected and sorted by fitness categories ranging from 0 to 11.

*Creation of the next generation:* To keep a constant population size, we randomly produce copies of males and females from the agent population if the number of daughters and sons is null or inferior to the population size. In the same way, we randomly remove daughters and sons if the number of daughters and sons produced is superior to the population size. Then, the parent traits are replaced by the offspring traits (i.e. male cognitive abilities and female selectivity and accuracy) and the other characteristics are reinitialized. These new agents represent the individuals for the next generation.

B. Code of the model

The code includes two parts. The first one contains the script for the simulation (*Main simulation script*, *General Parameters, Simulation, Class environment, Class Agent, ClassFoodPatch, ClassMale, ClassFemale)* and the second one the script for the analyze of data (*Main Data Analysis, Data Analysis).* This code was written for Python 3.6.

#### Main simulation script ####

import numpy
import time
import ScriptAnalyseData
import GeneralParameters as GenParam
import Simulation
import multiprocessing as mp
import os

def main(manager, pool):
 Time1 = time.time()

 # Creation of a data file
 GenParam.Populationfile = open('SimulationPopulationData.txt', 'w')
 GenParam.Populationfile.write("Range\tRepetition\tGeneration\tMoyPrecision\tMoyMateInitialCriterion\t"
 "SdPrecisionPopulation\tSdMateInitialCriterion\tMoyAbilityTerritories\t"
 "SdAbilityTerritories\n")

 # Run the simulation
 Simulation.RangeLoop(manager, pool)

 pool.close()
 pool.join()
 Time2 = time.time()

 # Close the file
 GenParam.Populationfile.close()

 # Check how many times no one mated in a given simulation
 if Simulation.CountNbOfBreakMale == 0 and Simulation.CountNbOfBreakFemale == 0:
 ScriptAnalyseData.AnalyseDataReady = "Yes"
 print("CountNbOfBreakMale", Simulation.CountNbOfBreakMale,
 "CountNbOfBreakFemale", Simulation.CountNbOfBreakFemale)

 # Save the datafiles
 with open('DATABRUTEAbilityValueByRangeByRepetitionByGeneration.npy', 'wb') as f:
 numpy.save(f, GenParam.AbilityValueByRangeByRepetitionByGeneration)
 with open('DATABRUTEMateChoiceCriterionValueByRangeByRepetitionByGeneration.npy', 'wb') as f:
 numpy.save(f, GenParam.MateChoiceCriterionValueByRangeByRepetitionByGeneration)
 with open('DATABRUTEPrecisionValueByRangeByRepetitionByGeneration.npy', 'wb') as f:
 numpy.save(f, GenParam.PrecisionValueByRangeByRepetitionByGeneration)
 with open('DATABRUTEFitnessByRangeByRepetitionByGenerationByAbility.npy', 'wb') as f:
 numpy.save(f, GenParam.FitnessByRangeByRepetitionByGenerationByAbility)
 with open('DATABRUTEFitnessByRangeByRepetitionByGenerationByMateChoiceCriterion.npy', 'wb') as f:
 numpy.save(f, GenParam.FitnessByRangeByRepetitionByGenerationByMateChoiceCriterion)
 with open('DATABRUTEFitnessByRangeByRepetitionByGenerationByPrecision.npy', 'wb') as f:
 numpy.save(f, GenParam.FitnessByRangeByRepetitionByGenerationByPrecision)
 with open('DATABRUTEReprodSuccessByRangeByRepetitionByGenerationByAbility.npy', 'wb') as f:
 numpy.save(f, GenParam.ReprodSuccessByRangeByRepetitionByGenerationByAbility)
 with open('DATABRUTEReprodSuccessByRangeByRepetitionByGenerationByMateChoiceCriterion.npy', 'wb') as f:
 numpy.save(f, GenParam.ReprodSuccessByRangeByRepetitionByGenerationByMateChoiceCriterion)
 with open('DATABRUTEReprodSuccessByRangeByRepetitionByGenerationByPrecision.npy', 'wb') as f:
 numpy.save(f, GenParam.ReprodSuccessByRangeByRepetitionByGenerationByPrecision)

 else:
 print("CountNbOfBreakMale", Simulation.CountNbOfBreakMale,
 "CountNbOfBreakFemale", Simulation.CountNbOfBreakFemale)
 Time3 = time.time()
 print("Total Time =", Time3-Time1, "Time Simulation", Time2-Time1, "Time Analyse", Time3-Time2)

if __name__=='__main__':
 manager = mp.Manager()
 pool = mp.Pool(1) # Number of repetitions that need to be run simultaneously
 print("cpu count:", os.cpu_count())
 main(manager, pool)

### General Parameters ###

import multiprocessing as mp

Range = mp.Value('i', 10)
FoodPatchNumber = mp.Value('i', 5)
IncreaseVariable = mp.Value('i', 20)

# Data files
malefile = None
femalefile = None
maleterritoryfile = None
Populationfile = None
DistributionAbilityMalefile = None

# Fixed variables
TotalNumberOfSimulation = 1000 # Number of generations
TotalNumberOfScenarioRepetition = 100 # Number of repetitions
MatrixSize = 14 # Size of the matrix long = large
CostCognitionFactor = 1 # Cost of male cognitive abilities
FertilityFactor = 1 # Female fertility
FemaleNumber = 100 # Number of females nF
MaleNumber = 100 # Number of females nM

# Manipulated variables
ForagingTimeStepNumber = 200 # Number of time steps in the foraging period
MateTimeStepNumber = 200 # Number of time Steps in the mating period
FoodPatchQuality = 100 # Number of food items contained in each patch
MaleAbilityToFindFood = 10 # Maximum number of time steps before a male find food
TimeStepNbCompleteInformation = 5 # Number of time steps needed for females to perfectly assess the male trait

# Lists including all data needed to run AnalyseData
AbilityValueByRangeByRepetitionByGeneration = []
MateChoiceCriterionValueByRangeByRepetitionByGeneration = []
PrecisionValueByRangeByRepetitionByGeneration = []
FitnessByRangeByRepetitionByGenerationByAbility = []
FitnessByRangeByRepetitionByGenerationByMateChoiceCriterion = []
FitnessByRangeByRepetitionByGenerationByPrecision = []
ReprodSuccessByRangeByRepetitionByGenerationByAbility = []
ReprodSuccessByRangeByRepetitionByGenerationByPrecision = []
ReprodSuccessByRangeByRepetitionByGenerationByMateChoiceCriterion = []

### Simulation ###

import GeneralParameters as GenParam
from functools import partial
import ClassFemale
import ClassMale
import ClassFoodPatch
import ClassEnvironment
import ClassAgentPopulation

sc = 0
r = 0
s = 0
CountNbOfBreakMale = 0
CountNbOfBreakFemale = 0

# Iterations over different environmental conditions
def RangeLoop(manager,pool):
 global sc
 for sc in range(GenParam.Range.value):
 print("RangeNb", sc)
 RepetitionLoop(manager,pool)


# Run several simulation simultaneously
def Parallelized_function(AbilityValueByRepetitionByGeneration,
 MateChoiceCriterionValueByRepetitionByGeneration,
 PrecisionValueByRepetitionByGeneration,
 FitnessByRepetitionByGenerationByAbility,
 FitnessByRepetitionByGenerationByMateChoiceCriterion,
 FitnessByRepetitionByGenerationByPrecision,
 ReprodSuccessByRepetitionByGenerationByAbility,
 ReprodSuccessByRepetitionByGenerationByPrecision,
 ReprodSuccessByRepetitionByGenerationByMateChoiceCriterion, iterable_item):
 # Creation of a population containing a list of males
 MalePopInstance = ClassAgentPopulation.AgentPopulation(ClassMale.Male, GenParam.MaleNumber)
 #print(id(MalePopInstance), " ID male pop intance")
 # Creation of a population containing a list of females
 FemalePopInstance = ClassAgentPopulation.AgentPopulation(ClassFemale.Female, GenParam.FemaleNumber)
 GenerationLoop(MalePopInstance,
 FemalePopInstance,
 AbilityValueByRepetitionByGeneration,
 MateChoiceCriterionValueByRepetitionByGeneration,
 PrecisionValueByRepetitionByGeneration,
 FitnessByRepetitionByGenerationByAbility,
 FitnessByRepetitionByGenerationByMateChoiceCriterion,
 FitnessByRepetitionByGenerationByPrecision,
 ReprodSuccessByRepetitionByGenerationByAbility,
 ReprodSuccessByRepetitionByGenerationByPrecision,
 ReprodSuccessByRepetitionByGenerationByMateChoiceCriterion)


def RepetitionLoop(manager,pool):
 global r
 AbilityValueByRepetitionByGeneration = manager.list()
 MateChoiceCriterionValueByRepetitionByGeneration = manager.list()
 PrecisionValueByRepetitionByGeneration = manager.list()
 FitnessByRepetitionByGenerationByAbility = manager.list()
 FitnessByRepetitionByGenerationByMateChoiceCriterion = manager.list()
 FitnessByRepetitionByGenerationByPrecision = manager.list()
 ReprodSuccessByRepetitionByGenerationByAbility = manager.list()
 ReprodSuccessByRepetitionByGenerationByPrecision = manager.list()
 ReprodSuccessByRepetitionByGenerationByMateChoiceCriterion = manager.list()

 func = partial(Parallelized_function,
 AbilityValueByRepetitionByGeneration,
 MateChoiceCriterionValueByRepetitionByGeneration,
 PrecisionValueByRepetitionByGeneration,
 FitnessByRepetitionByGenerationByAbility,
 FitnessByRepetitionByGenerationByMateChoiceCriterion,
 FitnessByRepetitionByGenerationByPrecision,
 ReprodSuccessByRepetitionByGenerationByAbility,
 ReprodSuccessByRepetitionByGenerationByPrecision,
 ReprodSuccessByRepetitionByGenerationByMateChoiceCriterion)
 results = pool.map(func, range(GenParam.TotalNumberOfScenarioRepetition))

 GenParam.FoodPatchNumber.value = GenParam.FoodPatchNumber.value + GenParam.IncreaseVariable.value

 GenParam.AbilityValueByRangeByRepetitionByGeneration.append(AbilityValueByRepetitionByGeneration)
 GenParam.MateChoiceCriterionValueByRangeByRepetitionByGeneration.append(MateChoiceCriterionValueByRepetitionByGeneration)
 GenParam.PrecisionValueByRangeByRepetitionByGeneration.append(PrecisionValueByRepetitionByGeneration)

 GenParam.FitnessByRangeByRepetitionByGenerationByAbility.append(FitnessByRepetitionByGenerationByAbility)
 GenParam.FitnessByRangeByRepetitionByGenerationByMateChoiceCriterion.append(FitnessByRepetitionByGenerationByMateChoiceCriterion)
 GenParam.FitnessByRangeByRepetitionByGenerationByPrecision.append(FitnessByRepetitionByGenerationByPrecision)

 GenParam.ReprodSuccessByRangeByRepetitionByGenerationByAbility. append(ReprodSuccessByRepetitionByGenerationByAbility)
 GenParam.ReprodSuccessByRangeByRepetitionByGenerationByPrecision.append(ReprodSuccessByRepetitionByGenerationByPrecision)
 GenParam.ReprodSuccessByRangeByRepetitionByGenerationByMateChoiceCriterion.append(ReprodSuccessByRepetitionByGenerationByMateChoiceCriterion)


def GenerationLoop(MalePopInstance,
 FemalePopInstance,
 AbilityValueByRepetitionByGeneration,
 MateChoiceCriterionValueByRepetitionByGeneration,
 PrecisionValueByRepetitionByGeneration,
 FitnessByRepetitionByGenerationByAbility,
 FitnessByRepetitionByGenerationByMateChoiceCriterion,
 FitnessByRepetitionByGenerationByPrecision,
 ReprodSuccessByRepetitionByGenerationByAbility,
 ReprodSuccessByRepetitionByGenerationByPrecision,
 ReprodSuccessByRepetitionByGenerationByMateChoiceCriterion):
 global s
 global CountNbOfBreakMale
 global CountNbOfBreakFemale

 FitnessByGenerationByAbility = [[] for x in range(GenParam.TotalNumberOfSimulation)]
 FitnessByGenerationByMateChoiceCriterion = [[] for x in range(GenParam.TotalNumberOfSimulation)]
 FitnessByGenerationByPrecision = [[] for x in range(GenParam.TotalNumberOfSimulation)]

 ReprodSuccessByGenerationByAbility = [[] for x in range(GenParam.TotalNumberOfSimulation)]
 ReprodSuccessByGenerationByPrecision = [[] for x in range(GenParam.TotalNumberOfSimulation)]
 ReprodSuccessByGenerationByMateChoiceCriterion = [[] for x in range(GenParam.TotalNumberOfSimulation)]

 AbilityValueByGeneration = [[] for x in range(GenParam.TotalNumberOfSimulation)]
 MateChoiceCriterionValueByGeneration = [[] for x in range(GenParam.TotalNumberOfSimulation)]
 PrecisionValueByGeneration = [[] for x in range(GenParam.TotalNumberOfSimulation)]

 for s in range(GenParam.TotalNumberOfSimulation):
 # Create the foraging environment
 #print("Generation nb:", s)
 ForagingEnvironmentInstance = ClassEnvironment.Environment() # Creation of an environment with of patches
 ForagingEnvironmentInstance.CharacterizeTerritory(ClassFoodPatch.FoodPatch, GenParam.FoodPatchNumber.value)
 ForagingEnvironmentInstance.setInitialPositionTerritoryList() # Set the localization of each patch

 # Male Foraging period
 MaleSocialForagingTimeLoop(MalePopInstance, ForagingEnvironmentInstance)

 # Create the mating environment
 MateChoiceEnvironmentInstance = ClassEnvironment.Environment() # Creation of an environment containing mate
 MateChoiceEnvironmentInstance.CharacterizeTerritoryWithList(MalePopInstance.AgentList) # Populate by males
 MateChoiceEnvironmentInstance.AppliedCostCognitionToFitness() # Calculate the foraging success of each male
 MateChoiceEnvironmentInstance.setInitialPositionTerritoryList() # Set the localization of each male

 # Female Mating period
 MateChoiceTimeLoop(FemalePopInstance, MateChoiceEnvironmentInstance)

 # List of males' and females' foraging success
 # Males
 FitnessByAbilityList = [[] for x in range(GenParam.MaleAbilityToFindFood+1)]
 #print(id(FitnessByAbilityList), "ID fitness by abilitylist")
 MateChoiceEnvironmentInstance.FitnessByAbility(FitnessByAbilityList)
 #print(FitnessByAbilityList, "FitnessByAbilityList")
 FitnessByGenerationByAbility[s].append(FitnessByAbilityList)

 # Females
 FitnessByMateChoiceCriterionList = [[] for x in range(GenParam.MaleAbilityToFindFood+1)]
 FemalePopInstance.FitnessByMateChoiceCriterion(FitnessByMateChoiceCriterionList)
 FitnessByGenerationByMateChoiceCriterion[s].append(FitnessByMateChoiceCriterionList)
 FitnessByPrecisionList = [[] for x in range(GenParam.TimeStepNbCompleteInformation+1)]
 FemalePopInstance.FitnessByPrecision(FitnessByPrecisionList)
 FitnessByGenerationByPrecision[s].append(FitnessByPrecisionList)

 # Is Anyone Mated?
 MateChoiceEnvironmentInstance.IsAnyoneMated() # break if no one is mated
 if MateChoiceEnvironmentInstance.AnyoneMated == False:
 print("Aucun mâle apparié")
 CountNbOfBreakMale = CountNbOfBreakMale + 1
 break
 FemalePopInstance.IsAnyoneMated()
 if FemalePopInstance.AnyoneMated == False:
 print("Aucune femelle appariée")
 CountNbOfBreakFemale = CountNbOfBreakFemale + 1
 break

 CalculateMeanTrait(FemalePopInstance, MateChoiceEnvironmentInstance)
 CalculateFitnessTrait(FemalePopInstance, MateChoiceEnvironmentInstance)

 # List of males' and females' reproductive success
 ReprodSuccessByAbilityList = [[] for x in range(GenParam.MaleAbilityToFindFood + 1)]
 ReprodSuccessByPrecisionList = [[] for x in range(GenParam.TimeStepNbCompleteInformation + 1)]
 ReprodSuccessByMateChoiceCriterionList = [[] for x in range(GenParam.MaleAbilityToFindFood + 1)]
 # Production of offspring
 CreationOfNextGenerationWithHeritableTrait(FemalePopInstance,
 MateChoiceEnvironmentInstance,
 MalePopInstance,
 ReprodSuccessByAbilityList,
 ReprodSuccessByPrecisionList,
 ReprodSuccessByMateChoiceCriterionList)

 ReprodSuccessByGenerationByAbility[s].append(ReprodSuccessByAbilityList)
 ReprodSuccessByGenerationByPrecision[s].append(ReprodSuccessByPrecisionList)
 ReprodSuccessByGenerationByMateChoiceCriterion[s].append(ReprodSuccessByMateChoiceCriterionList)
 AbilityValueByGeneration[s].append(MalePopInstance.AbilityValueByRange())
 MateChoiceCriterionValueByGeneration[s].append(FemalePopInstance.MateChoiceCriterionValueByRange())
 PrecisionValueByGeneration[s].append(FemalePopInstance.PrecisionValueByRange())
 #print('Current Process:', mp.current_process().name)

 FitnessByRepetitionByGenerationByAbility.append(FitnessByGenerationByAbility)
 FitnessByRepetitionByGenerationByMateChoiceCriterion.append(FitnessByGenerationByMateChoiceCriterion)
 FitnessByRepetitionByGenerationByPrecision.append(FitnessByGenerationByPrecision)
 ReprodSuccessByRepetitionByGenerationByAbility.append(ReprodSuccessByGenerationByAbility)
 ReprodSuccessByRepetitionByGenerationByPrecision.append(ReprodSuccessByGenerationByPrecision)
 ReprodSuccessByRepetitionByGenerationByMateChoiceCriterion.append(ReprodSuccessByGenerationByMateChoiceCriterion)
 AbilityValueByRepetitionByGeneration.append(AbilityValueByGeneration)
 MateChoiceCriterionValueByRepetitionByGeneration.append(MateChoiceCriterionValueByGeneration)
 PrecisionValueByRepetitionByGeneration.append(PrecisionValueByGeneration)


def MaleSocialForagingTimeLoop(MalePopInstance, ForagingEnvironmentInstance):
 for ft in range(GenParam.ForagingTimeStepNumber):
 # Update localization of males + check if they are on a food patch
 MalePopInstance.UpdatePositionAgentPopulation(ForagingEnvironmentInstance.TerritoryList)
 # MalePopInstance.PrintAllAgentParameter()
 # ForagingEnvironmentInstance.PrintAllTerritoryParameter()
 # Males consume the food
 MalePopInstance.PopulationActionWhenAgentIsOverTerritory(ForagingEnvironmentInstance.TerritoryList)
 #MalePopInstance.SaveAllAgentParameterToFile(sc, r, s, ft, GenParam.malefile) # Save data in the file


def MateChoiceTimeLoop(FemalePopInstance, MateChoiceEnvironmentInstance):
 global sc
 global r
 global s
 for mt in range(GenParam.MateTimeStepNumber):
 # Update females' localization + check if they are over a male territory
 FemalePopInstance.UpdatePositionAgentPopulation(MateChoiceEnvironmentInstance.TerritoryList)
 # Estimate the male trait + decide to mate or not
 FemalePopInstance.PopulationActionWhenAgentIsOverTerritory(MateChoiceEnvironmentInstance.TerritoryList)


def CalculateMeanTrait(FemalePopInstance, MateChoiceEnvironmentInstance):
 global sc
 global r
 global s
 FemalePopInstance.CalculateSaveMoyTraitPopulation(sc, r, s, GenParam.Populationfile)
 MateChoiceEnvironmentInstance.CalculateSaveMoyAbilityToFindFoodTerritories(GenParam.Populationfile)


def CalculateFitnessTrait(FemalePopInstance, MateChoiceEnvironmentInstance):
 StatFitnessMale = MateChoiceEnvironmentInstance.CalculateStatFitnessPopulation()
 StatFitnessFemale = FemalePopInstance.CalculateStatFitnessPopulation()
 # Assign individuals a fitness category
 MateChoiceEnvironmentInstance.PopAttributionFitnessCategory(StatFitnessMale)
 FemalePopInstance.PopAttributionFitnessCategory(StatFitnessFemale)


def CreationOfNextGenerationWithHeritableTrait(FemalePopInstance,
 MateChoiceEnvironmentInstance,
 MalePopInstance,ReprodSuccessByAbilityList,
 ReprodSuccessByPrecisionList,
 ReprodSuccessByMateChoiceCriterionList):
 # Production of sons and daughters
 PossibleValueOfAbilityToFindFood = MateChoiceEnvironmentInstance.CreationListHeritableTrait()
 MatedFemalePrecisionMatedCriterion = FemalePopInstance.CreationListHeritableTrait()
 # print("List of abilities", PossibleValueOfAbilityToFindFood,
 # "List of Precision and Criterion", MatedFemalePrecisionMatedCriterion
 FemalePopInstance.ReprodSuccessByPrecision(ReprodSuccessByPrecisionList)
 FemalePopInstance.ReprodSuccessByMateChoiceCriterion(ReprodSuccessByMateChoiceCriterionList)
 MateChoiceEnvironmentInstance.ReprodSuccessByAbility(ReprodSuccessByAbilityList)

 # Replace fathers by sons and mothers by daughters for the next generation
 MalePopInstance.ReinitializeAgentPopulation(PossibleValueOfAbilityToFindFood)
 #print("male ability new generation", MalePopInstance.AgentList[0].AbilityToFindFood,
 # "Time to access food", MalePopInstance.AgentList[0].TimeBeforeAccessFood,
 # "quality:", MalePopInstance.AgentList[0].Quality)
 FemalePopInstance.ReinitializeAgentPopulation(MatedFemalePrecisionMatedCriterion)
 # print("Precision new generation", FemalePopInstance.AgentList[0].Precision,
 # "Criterion new generation", FemalePopInstance.AgentList[0].MateInitialCriterion
 list.clear(PossibleValueOfAbilityToFindFood)
 list.clear(MatedFemalePrecisionMatedCriterion[0])
 list.clear(MatedFemalePrecisionMatedCriterion[1])

## Class Environment ##

import random
import GeneralParameters as GenParam
import numpy as np
from itertools import repeat

class Environment:
 def __init__(self):
 self.TerritoryList = []
 self.AnyoneMated = False

 def CharacterizeTerritory(self, EnvironmentType, EnvironmentNumber):
 for EnvironmentIndex in range(EnvironmentNumber):
 self.TerritoryList.append(EnvironmentType(EnvironmentIndex + 1))


 def CharacterizeTerritoryWithList(self, TerritoryList):
 self.TerritoryList = TerritoryList


 def AppliedCostCognitionToFitness(self):
 for TerritoryInstance in self.TerritoryList:
 #print("AbilityToFindFood", TerritoryInstance.AbilityToFindFood,
 # "Quality", TerritoryInstance.Quality, "CostCogn", TerritoryInstance.CostCognition)
 if TerritoryInstance.Quality <= (GenParam.CostCognitionFactor*TerritoryInstance.CostCognition):
 TerritoryInstance.Quality = 0
 #print("Quality", TerritoryInstance.Quality)
 else:
 TerritoryInstance.Quality = TerritoryInstance.Quality - \
 (GenParam.CostCognitionFactor*TerritoryInstance.CostCognition)
 #print("CostCognitionFactor", GenParam.CostCognitionFactor,
 # "CostCognition", TerritoryInstance.CostCognition, "Quality after CostCogn", TerritoryInstance.Quality)


 def setInitialPositionTerritoryList(self): # Unique and random position
 Coordinate = [(random.randint(0, GenParam.MatrixSize-1),
 (random.randint(0, GenParam.MatrixSize-1))) for _ in range(len(self.TerritoryList))]
 UniqueCoordinate = list(set(Coordinate))

 while (len(UniqueCoordinate) < len(self.TerritoryList)):
 UniqueCoordinate.append((random.randint(0, GenParam.MatrixSize-1),
 (random.randint(0, GenParam.MatrixSize-1))))
 UniqueCoordinate = list(set(UniqueCoordinate))

 for TerritoryInstance in self.TerritoryList:
 TerritoryInstance.Position = list(UniqueCoordinate[TerritoryInstance.ID - 1])


 def getAllTerritoryPosition(self):
 AllTerritoryPosition = []
 for TerritoryInstance in self.TerritoryList:
 AllTerritoryPosition.append(TerritoryInstance.Position)
 return AllTerritoryPosition


 def IsAnyoneMated(self):
 NumberTerritoryMated = 0
 for TerritoryInstance in self.TerritoryList:
 #print("Male ID", TerritoryInstance.ID, "is available", TerritoryInstance.Availability)

 if TerritoryInstance.Availability == False:
 NumberTerritoryMated = NumberTerritoryMated + 1
 #print("NumberTerritoryMated", NumberTerritoryMated)
 self.AnyoneMated = True
 #print("AnyoneMated", self.AnyoneMated)

 if NumberTerritoryMated == 0:
 self.AnyoneMated = False


 def CalculateSaveMoyAbilityToFindFoodTerritories(self, Populationfile):
 AbilityTerritories = []
 #print("AbilityTerritoriesBefore", AbilityTerritories)

 for TerritoryInstance in self.TerritoryList:
 AbilityTerritories.append(TerritoryInstance.AbilityToFindFood)
 MoyAbilityTerritories = sum(AbilityTerritories) / len(AbilityTerritories)
 #print("MoyAbilityTerritories", MoyAbilityTerritories)
 SdAbilityTerritories = sum([((x - MoyAbilityTerritories) ** 2)
 for x in AbilityTerritories]) / len(AbilityTerritories)
 #print("SdAbilityTerritories", SdAbilityTerritories)
 #Populationfile.write(" "+str(MoyAbilityTerritories)+" "+str(SdAbilityTerritories)+"\n")


 def CalculateStatFitnessPopulation(self):
 FitnessPop = []
 FitnessPopPositiveQuality = []
 for TerritoryInstance in self.TerritoryList:
 FitnessPop.append(TerritoryInstance.Quality)
 for IndexQuality in FitnessPop:
 if IndexQuality > 0:
 FitnessPopPositiveQuality.append(IndexQuality)
 #print("FitnessPopListMale", FitnessPopPositiveQuality)
 if len(FitnessPopPositiveQuality) > 0:
 Q1FitnessMale = np.percentile(FitnessPopPositiveQuality, 25, interpolation='midpoint')
 MoyFitnessMale = sum(FitnessPopPositiveQuality) / len(FitnessPopPositiveQuality)
 Q2FitnessMale = np.percentile(FitnessPopPositiveQuality, 75, interpolation='midpoint')
 MaxFitnessMale = max(FitnessPopPositiveQuality)
 else :
 Q1FitnessMale = 0
 MoyFitnessMale = 0
 Q2FitnessMale = 0
 MaxFitnessMale =0
 return Q1FitnessMale, MoyFitnessMale, Q2FitnessMale, MaxFitnessMale


 def PopAttributionFitnessCategory(self, StatFitnessMale):
 for TerritoryInstance in self.TerritoryList:
 TerritoryInstance.AttributionFitnessCategory(StatFitnessMale)


 def CreationListHeritableTrait(self):
 SonAbility = []
 for TerritoryInstance in self.TerritoryList:
 if TerritoryInstance.Availability == False and TerritoryInstance.FitnessCategory != 0:

 if TerritoryInstance.Availability == False and TerritoryInstance.FitnessCategory == 1:
 SonAbility.extend(repeat(TerritoryInstance.AbilityToFindFood, 1 * GenParam.FertilityFactor))
 TerritoryInstance.ReproductiveSuccess = TerritoryInstance.ReproductiveSuccess + \
 (1 * GenParam.FertilityFactor)
 elif TerritoryInstance.Availability == False and TerritoryInstance.FitnessCategory == 2:
 SonAbility.extend(repeat(TerritoryInstance.AbilityToFindFood, 2 * GenParam.FertilityFactor))
 TerritoryInstance.ReproductiveSuccess = TerritoryInstance.ReproductiveSuccess + \
 (2 * GenParam.FertilityFactor)

 elif TerritoryInstance.Availability == False and TerritoryInstance.FitnessCategory == 3:
 SonAbility.extend(repeat(TerritoryInstance.AbilityToFindFood, 3 * GenParam.FertilityFactor))
 TerritoryInstance.ReproductiveSuccess = TerritoryInstance.ReproductiveSuccess + \
 (3 * GenParam.FertilityFactor)

 elif TerritoryInstance.Availability == False and TerritoryInstance.FitnessCategory == 4:
 SonAbility.extend(repeat(TerritoryInstance.AbilityToFindFood, 4 * GenParam.FertilityFactor))
 TerritoryInstance.ReproductiveSuccess = TerritoryInstance.ReproductiveSuccess + \
 (4 * GenParam.FertilityFactor)

 elif TerritoryInstance.Availability == False and TerritoryInstance.FitnessCategory == 5:
 SonAbility.extend(repeat(TerritoryInstance.AbilityToFindFood, 5 * GenParam.FertilityFactor))
 TerritoryInstance.ReproductiveSuccess = TerritoryInstance.ReproductiveSuccess + \
 (5 * GenParam.FertilityFactor)

 elif TerritoryInstance.Availability == False and TerritoryInstance.FitnessCategory == 6:
 SonAbility.extend(repeat(TerritoryInstance.AbilityToFindFood, 6 * GenParam.FertilityFactor))
 TerritoryInstance.ReproductiveSuccess = TerritoryInstance.ReproductiveSuccess + \
 (6 * GenParam.FertilityFactor)

 elif TerritoryInstance.Availability == False and TerritoryInstance.FitnessCategory == 7:
 SonAbility.extend(repeat(TerritoryInstance.AbilityToFindFood, 7 * GenParam.FertilityFactor))
 TerritoryInstance.ReproductiveSuccess = TerritoryInstance.ReproductiveSuccess + \
 (7 * GenParam.FertilityFactor)

 elif TerritoryInstance.Availability == False and TerritoryInstance.FitnessCategory == 8:
 SonAbility.extend(repeat(TerritoryInstance.AbilityToFindFood, 8 * GenParam.FertilityFactor))
 TerritoryInstance.ReproductiveSuccess = TerritoryInstance.ReproductiveSuccess + \
 (8 * GenParam.FertilityFactor)

 elif TerritoryInstance.Availability == False and TerritoryInstance.FitnessCategory == 9:
 SonAbility.extend(repeat(TerritoryInstance.AbilityToFindFood, 9 * GenParam.FertilityFactor))
 TerritoryInstance.ReproductiveSuccess = TerritoryInstance.ReproductiveSuccess + \
 (9 * GenParam.FertilityFactor)

 elif TerritoryInstance.Availability == False and TerritoryInstance.FitnessCategory == 10:
 SonAbility.extend(repeat(TerritoryInstance.AbilityToFindFood, 10 * GenParam.FertilityFactor))
 TerritoryInstance.ReproductiveSuccess = TerritoryInstance.ReproductiveSuccess +\
 (10 * GenParam.FertilityFactor)

 elif TerritoryInstance.Availability == False and TerritoryInstance.FitnessCategory == 11:
 SonAbility.extend(repeat(TerritoryInstance.AbilityToFindFood, 11 * GenParam.FertilityFactor))
 TerritoryInstance.ReproductiveSuccess = TerritoryInstance.ReproductiveSuccess + \
 (11 * GenParam.FertilityFactor)
 else:
 raise RuntimeError("error in Reproductive success attribution Male")

 if len(SonAbility) == 0:
 while len(SonAbility) < GenParam.MaleNumber:
 RandomTerritoryInstance = random.choice(self.TerritoryList)
 SonAbility.append(RandomTerritoryInstance.AbilityToFindFood)
 while len(SonAbility) < GenParam.MaleNumber:
 RandomItemFromListCrit = random.choice(SonAbility)
 SonAbility.append(RandomItemFromListCrit)
 while len(SonAbility) > GenParam.MaleNumber:
 RandomItemFromListCrit = random.choice(SonAbility)
 SonAbility.remove(RandomItemFromListCrit)
 return SonAbility


 def getAllTerritoryQuality(self):
 AllTerritoryQuality = []
 for TerritoryInstance in self.TerritoryList:
 AllTerritoryQuality.append(TerritoryInstance.Quality)
 return AllTerritoryQuality


 def PrintAllTerritoryParameter(self):
 for TerritoryInstance in self.TerritoryList:
 print(TerritoryInstance)


 def SaveAllTerritoryParameterToFile(self, Range, Repetition, SimulationNb, TimeStep, Territoryfile):
 for TerritoryInstance in self.TerritoryList:
 Territoryfile.write(str(Range)+"\t\t"+
 str(Repetition)+"\t\t"+
 str(SimulationNb) + "\t\t" +
 str(TimeStep) + "\t\t" +
 str(TerritoryInstance))


 def FitnessByAbility(self, FitnessByAbilityList):
 for TerritoryInstance in self.TerritoryList:
 FitnessByAbilityList[TerritoryInstance.AbilityToFindFood].append(TerritoryInstance.Quality)
 #print("male num", TerritoryInstance.ID,
 # " with ability",TerritoryInstance.AbilityToFindFood, "and quality", TerritoryInstance.Quality)


 def ReprodSuccessByAbility(self, ReprodSuccessByAbilityList):
 for TerritoryInstance in self.TerritoryList:
 ReprodSuccessByAbilityList[TerritoryInstance.AbilityToFindFood].append(TerritoryInstance.ReproductiveSuccess)

## Class Agent ##

import random
import GeneralParameters as GenParam
import numpy as np
from itertools import repeat
import math

class AgentPopulation:
 def __init__(self, AgentType, AgentNumber):
 self.AgentList = []
 self.CharacterizeAgentPopulation(AgentType, AgentNumber)
 self.AnyoneMated = False


 def CharacterizeAgentPopulation(self, AgentType, AgentNumber):
 for AgentIndex in range(AgentNumber):
 self.AgentList.append(AgentType(AgentIndex + 1))


 def UpdatePositionAgentPopulation(self, TerritoryList):
 for AgentInstance in self.AgentList:
 AgentInstance.UpdatePosition(TerritoryList)
 AgentInstance.IsAgentOverTerritory(TerritoryList)


 def getAllAgentPosition(self):
 AllAgentPosition = []
 for AgentInstance in self.AgentList:
 AllAgentPosition.append(AgentInstance.Position)
 return AllAgentPosition


 def PopulationActionWhenAgentIsOverTerritory(self, TerritoryList):
 for AgentInstance in self.AgentList:
 if AgentInstance.AgentOverTerritory == True:
 #print("True")
 AgentInstance.ActionWhenAgentIsOverTerritory(TerritoryList)


 def AttributeAgentPopulationWithMalePosition(self,TerritoryList):
 for AgentInstance in self.AgentList:
 AgentInstance.AttributeMalePosition(TerritoryList)


 def IsAnyoneMated(self):
 NumberAgentMated = 0
 for AgentInstance in self.AgentList:
 #print("Female ID", AgentInstance.ID, "is mated", AgentInstance.FoundMate)

 if AgentInstance.FoundMate == True:
 NumberAgentMated = NumberAgentMated + 1
 #print("NumberAgentMated", NumberAgentMated)
 self.AnyoneMated = True
 #print("AnyoneMated", self.AnyoneMated)

 if NumberAgentMated == 0:
 self.AnyoneMated = False


 def CalculateSaveMoyTraitPopulation(self, Scenario, Repetition, SimulationNb, Populationfile):
 PrecisionPopulation = []
 MateInitialCriterionPopulation = []
 MoyPrecisionPopulation = 0
 MoyMateInitialCriterionPopulation = 0
 SdPrecisionPopulation = 0
 SdMateInitialCriterionPopulation = 0
 #print("PrecisionPopulationBefore", PrecisionPopulation)
 for AgentInstance in self.AgentList:
 PrecisionPopulation.append(AgentInstance.Precision)
 MateInitialCriterionPopulation.append(AgentInstance.MateInitialCriterion)
 MoyPrecisionPopulation = sum(PrecisionPopulation)/ len(PrecisionPopulation)
 #print("MoyPrecisionPopulation", MoyPrecisionPopulation)
 MoyMateInitialCriterionPopulation = sum(MateInitialCriterionPopulation)/len(MateInitialCriterionPopulation)
 #print("MoyMateInitialCriterionPopulation", MoyMateInitialCriterionPopulation)
 SdPrecisionPopulation = math.sqrt(sum([((x - MoyPrecisionPopulation) ** 2) for x in PrecisionPopulation]) / len(PrecisionPopulation))
 #print("SdPrecisionPopulation", SdPrecisionPopulation)
 SdMateInitialCriterionPopulation = math.sqrt(sum([((x - MoyMateInitialCriterionPopulation) ** 2) for x in MateInitialCriterionPopulation])\
 / len(MateInitialCriterionPopulation))
 #print("SdMateInitialCriterionPopulation", SdMateInitialCriterionPopulation)
 #Populationfile.write(str(Scenario)+" "+str(Repetition)+" "+str(SimulationNb)+" "+str(MoyPrecisionPopulation)+
 # " "+str(MoyMateInitialCriterionPopulation)+" "+str(SdPrecisionPopulation)+" "
 # +str(SdMateInitialCriterionPopulation))

 #def CalculateSaveDataFitnessPrecisionPopulation(self):
 # FitnessPop = []
 # for AgentInstance in self.AgentList:
 # FitnessPop.append(AgentInstance.Quality)


 def CalculateStatFitnessPopulation(self):
 FitnessPop = []
 FitnessPopPositiveQuality = []
 for AgentInstance in self.AgentList:
 FitnessPop.append(AgentInstance.Quality)
 for IndexQuality in FitnessPop:
 if IndexQuality > 0:
 FitnessPopPositiveQuality.append(IndexQuality)
 # print("FitnessPopListfemale", FitnessPopPositiveQuality)
 if len(FitnessPopPositiveQuality) > 0:
 Q1FitnessFemale = np.percentile(FitnessPopPositiveQuality, 25, interpolation='midpoint')
 MoyFitnessFemale = sum(FitnessPopPositiveQuality) / len(FitnessPopPositiveQuality)
 Q2FitnessFemale = np.percentile(FitnessPopPositiveQuality, 75, interpolation='midpoint')
 MaxFitnessFemale = max(FitnessPopPositiveQuality)
 else :
 Q1FitnessFemale = 0
 MoyFitnessFemale = 0
 Q2FitnessFemale = 0
 MaxFitnessFemale = 0
 return Q1FitnessFemale, MoyFitnessFemale, Q2FitnessFemale, MaxFitnessFemale


 def PopAttributionFitnessCategory(self, StatFitnessFemale):
 for AgentInstance in self.AgentList:
 AgentInstance.AttributionFitnessCategory(StatFitnessFemale)


 def CreationListHeritableTrait(self):
 DaughterPrecision = []
 DaughterCriterion = []
 for AgentInstance in self.AgentList:
 if AgentInstance.FoundMate == True and AgentInstance.FitnessCategory != 0:

 if AgentInstance.FoundMate == True and AgentInstance.FitnessCategory == 1:
 DaughterPrecision.extend(repeat(AgentInstance.Precision, 1 * GenParam.FertilityFactor))
 #print("List of daughters precision", DaughterPrecision)
 DaughterCriterion.extend(repeat(AgentInstance.MateInitialCriterion, 1 * GenParam.FertilityFactor))
 # print("List of daughters selectivity", DaughterCriterion)
 AgentInstance.ReproductiveSuccess = AgentInstance.ReproductiveSuccess + \
 (1 * GenParam.FertilityFactor)

 elif AgentInstance.FoundMate == True and AgentInstance.FitnessCategory == 2:
 DaughterPrecision.extend(repeat(AgentInstance.Precision, 2 * GenParam.FertilityFactor))
 #print("List of daughters precision", DaughterPrecision)
 DaughterCriterion.extend(repeat(AgentInstance.MateInitialCriterion, 2 * GenParam.FertilityFactor))
 # print("List of daughters selectivity", DaughterCriterion)
 AgentInstance.ReproductiveSuccess = AgentInstance.ReproductiveSuccess + \
 (2 * GenParam.FertilityFactor)

 elif AgentInstance.FoundMate == True and AgentInstance.FitnessCategory == 3:
 DaughterPrecision.extend(repeat(AgentInstance.Precision, 3 * GenParam.FertilityFactor))
 #print("List of daughters precision", DaughterPrecision)
 DaughterCriterion.extend(repeat(AgentInstance.MateInitialCriterion, 3 * GenParam.FertilityFactor))
 # print("List of daughters selectivity", DaughterCriterion)
 AgentInstance.ReproductiveSuccess = AgentInstance.ReproductiveSuccess + \
 (3 * GenParam.FertilityFactor)

 elif AgentInstance.FoundMate == True and AgentInstance.FitnessCategory == 4:
 DaughterPrecision.extend(repeat(AgentInstance.Precision, 4 * GenParam.FertilityFactor))
 #print("List of daughters precision", DaughterPrecision)
 DaughterCriterion.extend(repeat(AgentInstance.MateInitialCriterion, 4 * GenParam.FertilityFactor))
 # print("List of daughters selectivity", DaughterCriterion)
 AgentInstance.ReproductiveSuccess = AgentInstance.ReproductiveSuccess + \
 (4 * GenParam.FertilityFactor)

 elif AgentInstance.FoundMate == True and AgentInstance.FitnessCategory == 5:
 DaughterPrecision.extend(repeat(AgentInstance.Precision, 5 * GenParam.FertilityFactor))
 # print("List of daughters precision", DaughterPrecision)
 DaughterCriterion.extend(repeat(AgentInstance.MateInitialCriterion, 5 * GenParam.FertilityFactor))
 # print("List of daughters selectivity", DaughterCriterion)
 AgentInstance.ReproductiveSuccess = AgentInstance.ReproductiveSuccess + \
 (5 * GenParam.FertilityFactor)

 elif AgentInstance.FoundMate == True and AgentInstance.FitnessCategory == 6:
 DaughterPrecision.extend(repeat(AgentInstance.Precision, 6 * GenParam.FertilityFactor))
 # print("List of daughters precision", DaughterPrecision)
 DaughterCriterion.extend(repeat(AgentInstance.MateInitialCriterion, 6 * GenParam.FertilityFactor))
 # print("List of daughters selectivity", DaughterCriterion)
 AgentInstance.ReproductiveSuccess = AgentInstance.ReproductiveSuccess + \
 (6 * GenParam.FertilityFactor)

 elif AgentInstance.FoundMate == True and AgentInstance.FitnessCategory == 7:
 DaughterPrecision.extend(repeat(AgentInstance.Precision, 7 * GenParam.FertilityFactor))
 # print("List of daughters precision", DaughterPrecision)
 DaughterCriterion.extend(repeat(AgentInstance.MateInitialCriterion, 7 * GenParam.FertilityFactor))
 # print("List of daughters selectivity", DaughterCriterion)
 AgentInstance.ReproductiveSuccess = AgentInstance.ReproductiveSuccess + \
 (7 * GenParam.FertilityFactor)

 elif AgentInstance.FoundMate == True and AgentInstance.FitnessCategory == 8:
 DaughterPrecision.extend(repeat(AgentInstance.Precision, 8 * GenParam.FertilityFactor))
 # print("List of daughters precision", DaughterPrecision)
 DaughterCriterion.extend(repeat(AgentInstance.MateInitialCriterion, 8 * GenParam.FertilityFactor))
 # print("List of daughters selectivity", DaughterCriterion)
 AgentInstance.ReproductiveSuccess = AgentInstance.ReproductiveSuccess + \
 (8 * GenParam.FertilityFactor)

 elif AgentInstance.FoundMate == True and AgentInstance.FitnessCategory == 9:
 DaughterPrecision.extend(repeat(AgentInstance.Precision, 9 * GenParam.FertilityFactor))
 # print("List of daughters precision", DaughterPrecision)
 DaughterCriterion.extend(repeat(AgentInstance.MateInitialCriterion, 9 * GenParam.FertilityFactor))
 # print("List of daughters selectivity", DaughterCriterion)
 AgentInstance.ReproductiveSuccess = AgentInstance.ReproductiveSuccess + \
 (9 * GenParam.FertilityFactor)

 elif AgentInstance.FoundMate == True and AgentInstance.FitnessCategory == 10:
 DaughterPrecision.extend(repeat(AgentInstance.Precision, 10 * GenParam.FertilityFactor))
 # print("List of daughters precision", DaughterPrecision)
 DaughterCriterion.extend(repeat(AgentInstance.MateInitialCriterion, 10 * GenParam.FertilityFactor))
 # print("List of daughters selectivity", DaughterCriterion)
 AgentInstance.ReproductiveSuccess = AgentInstance.ReproductiveSuccess + \
 (10 * GenParam.FertilityFactor)

 elif AgentInstance.FoundMate == True and AgentInstance.FitnessCategory == 11:
 DaughterPrecision.extend(repeat(AgentInstance.Precision, 11 * GenParam.FertilityFactor))
 # print("List of daughters precision", DaughterPrecision)
 DaughterCriterion.extend(repeat(AgentInstance.MateInitialCriterion, 11 * GenParam.FertilityFactor))
 # print("List of daughters selectivity", DaughterCriterion)
 AgentInstance.ReproductiveSuccess = AgentInstance.ReproductiveSuccess +\
 (11 * GenParam.FertilityFactor)
 else:
 raise RuntimeError("error in Reproductive success attribution Female")

 if len(DaughterPrecision) == 0 and len(DaughterCriterion) == 0:
 while len(DaughterPrecision) < GenParam.FemaleNumber and len(DaughterCriterion) < GenParam.FemaleNumber:
 RandomAgentInstance = random.choice(self.AgentList)
 DaughterPrecision.append(RandomAgentInstance.Precision)
 DaughterCriterion.append(RandomAgentInstance.MateInitialCriterion)

 while len(DaughterPrecision) < GenParam.FemaleNumber and len(DaughterCriterion) < GenParam.FemaleNumber:
 #print("length list of daughters precision", len(DaughterPrecision),
 # "and length list of daughters selectivity", len(DaughterCriterion))
 RandomItemFromListCrit = random.choice(DaughterCriterion)
 DaughterCriterion.append(RandomItemFromListCrit)
 RandomItemFromListPrec = random.choice(DaughterPrecision)
 DaughterPrecision.append(RandomItemFromListPrec)
 #print("RandomItemFromListPrec", RandomItemFromListPrec)
 #print("list of daughters precision", DaughterPrecision)
 #print("length", len(DaughterPrecision))

 while len(DaughterPrecision) > GenParam.FemaleNumber and len(DaughterCriterion) > GenParam.FemaleNumber:
 #print("length list daughter precision", len(DaughterPrecision),
 # "and length list daughterCriterion",len(DaughterCriterion))
 RandomItemFromListCrit = random.choice(DaughterCriterion)
 DaughterCriterion.remove(RandomItemFromListCrit)
 RandomItemFromListPrec = random.choice(DaughterPrecision)
 DaughterPrecision.remove(RandomItemFromListPrec)
 #print("RandomItemFromListPrec", RandomItemFromListPrec)
 #print("list of daughters precision", DaughterPrecision)
 #print("length", len(DaughterPrecision))
 return DaughterPrecision, DaughterCriterion


 def ReinitializeAgentPopulation(self, ListHeritableTrait):
 for AgentInstance in self.AgentList:
 AgentInstance.EvolutionaryUpdateCharacteristic(ListHeritableTrait)


 def PrintAllAgentParameter (self):
 for AgentInstance in self.AgentList:
 print(AgentInstance)


 def SaveAllAgentParameterToFile(self, Range, Repetition, SimulationNb, TimeStep, Agentfile):
 for AgentInstance in self.AgentList:
 Agentfile.write(str(Range)+"\t\t"+
 str(Repetition)+"\t\t"+
 str(SimulationNb)+"\t\t"+
 str(TimeStep)+"\t\t"+
 str(AgentInstance))


 def MateChoiceCriterionValueByRange(self):
 MateChoiceCriterionValueList = []
 for AgentInstance in self.AgentList:
 MateChoiceCriterionValueList.append(AgentInstance.MateInitialCriterion)
 return MateChoiceCriterionValueList


 def AbilityValueByRange(self):
 AbilityValueByRangeList = []
 for AgentInstance in self.AgentList:
 AbilityValueByRangeList.append(AgentInstance.AbilityToFindFood)
 return AbilityValueByRangeList


 def PrecisionValueByRange(self, ):
 PrecisionValueList = []
 for AgentInstance in self.AgentList:
 PrecisionValueList.append(AgentInstance.Precision)
 return PrecisionValueList


 def FitnessByMateChoiceCriterion(self, FitnessByMateChoiceCriterionList):
 for AgentInstance in self.AgentList:
 FitnessByMateChoiceCriterionList[AgentInstance.MateInitialCriterion].append(AgentInstance.Quality)


 def FitnessByPrecision(self, FitnessByPrecisionList):
 for AgentInstance in self.AgentList:
 FitnessByPrecisionList[AgentInstance.Precision].append(AgentInstance.Quality)


 def ReprodSuccessByMateChoiceCriterion(self, ReprodSuccessByMateChoiceCriterionList):
 for AgentInstance in self.AgentList:
 ReprodSuccessByMateChoiceCriterionList[AgentInstance.MateInitialCriterion].append(AgentInstance.ReproductiveSuccess)


 def ReprodSuccessByPrecision(self, ReprodSuccessByPrecisionList):
 for AgentInstance in self.AgentList:
 ReprodSuccessByPrecisionList[AgentInstance.Precision].append(AgentInstance.ReproductiveSuccess)

# Class Food Patch #

import GeneralParameters as GenParam

class FoodPatch:
 def __init__(self, ID):
 self.ID = ID
 self.Position = []
 self.Quality = GenParam.FoodPatchQuality
 self.Availability = True


 def __str__(self):
 return 'FoodPatch(ID='+str(self.ID)+', Position='+str(self.Position)+\
 ', nombre item de nourriture disponible = '+str(self.Quality)+ ')'

# Class Male #

import random
import GeneralParameters as GenParam

class Male:
 def __init__(self, ID):
 self.ID = ID
 self.Position = []
 self.setInitialPosition()
 self.Quality = 0
 #self.setCognition() # Attribution of a cognitive ability score for the male instance
 self.AgentOverTerritory = False
 self.TerritoryVisited = ()
 self.AgentOverWhichIndexTerritory = 0
 self.Availability = True
 self.AbilityToFindFood = self.setInitialAbilityToFindFood()
 self.TimeBeforeAccessFood = 0
 self.FitnessCategory = 0
 self.ReproductiveSuccess = 0
 self.CostCognition = self.setInitialCostCognition()


 def __str__(self):
 return str(self.ID)+"\t\t"+str(self.Quality)+"\t\t"+str(self.AbilityToFindFood)+"\t\t"\
 +str(self.Availability)+"\t\t"+str(self.ReproductiveSuccess)+'\n'


 def setInitialPosition(self):
 self.Position = [random.randint(0, GenParam.MatrixSize - 1), random.randint(0, GenParam.MatrixSize - 1)]


 #Characterization of males when they are an AgentPop (during foraging period)
 # and an Environment component (during mating period) ###
 def setPosition(self, x, y):
 self.Position.clear()
 self.Position = [x, y]


 def setInitialAbilityToFindFood(self):
 return random.randint(0, GenParam.MaleAbilityToFindFood)


 def setInitialCostCognition(self):
 return (GenParam.MaleAbilityToFindFood - self.AbilityToFindFood)


 def AttributionFitnessCategory(self, StatFitnessMale):
 if self.Quality <= 0:
 self.FitnessCategory = 0
 elif 1 <= self.Quality <= 10:
 self.FitnessCategory = 1
 elif 11 <= self.Quality <= 20:
 self.FitnessCategory = 2
 elif 21 <= self.Quality <= 30:
 self.FitnessCategory = 3
 elif 31 <= self.Quality <= 40:
 self.FitnessCategory = 4
 elif 41 <= self.Quality <= 50:
 self.FitnessCategory = 5
 elif 51 <= self.Quality <= 60:
 self.FitnessCategory = 6
 elif 61 <= self.Quality <= 70:
 self.FitnessCategory = 7
 elif 71 <= self.Quality <= 80:
 self.FitnessCategory = 8
 elif 81 <= self.Quality <= 90:
 self.FitnessCategory = 9
 elif 91 <= self.Quality <= 100:
 self.FitnessCategory = 10
 elif 101 <= self.Quality:
 self.FitnessCategory = 11
 else:
 raise RuntimeError("error fitness Category male")
 #print("maleID", self.ID, "male fitness category", self.FitnessCategory)
 return self.FitnessCategory


 def EvolutionaryUpdateCharacteristic(self, ListHeritableTrait):
 IndexAbilityToFindFood = random.randint(0, len(ListHeritableTrait)-1)
 #print("IndexAbilityToFindFood", IndexAbilityToFindFood, "ListHeritableTrait", len(ListHeritableTrait))
 self.AbilityToFindFood = ListHeritableTrait.pop(IndexAbilityToFindFood)
 #print("ability to find food of the new generation", self.AbilityToFindFood,
 # "list of hertiable trait", ListHeritableTrait)
 self.setInitialPosition()
 self.Quality = 0
 self.AgentOverTerritory = False
 self.AgentOverWhichIndexTerritory = 0
 self.Availability = True
 self.TerritoryVisited = ()
 self.TimeBeforeAccessFood = 0
 self.FitnessCategory = 0
 self.ReproductiveSuccess = 0


 ### Characterization of males when they are an AgentPop component (During foraging period) ###
 def UpdatePosition(self, TerritoryList):
 if self.AgentOverTerritory == False:
 # Random Walk for males
 DirectionPosition = random.randint(1, 4) # create an object with 25% of chance to be chosen
 if DirectionPosition == 1: # if this object is equivalent to 1
 self.Position[0] += -1 # Move the given female to the left to one unit
 if self.Position[0] < 0: # if the updated Position[0] is outboard the matrix
 self.Position[0] = (GenParam.MatrixSize - 1) # Move the female to the other side of the matrix
 elif DirectionPosition == 2: # if this object is equivalent to 2
 self.Position[0] += 1 # Move the given female to the right to one unit
 if self.Position[0] > (GenParam.MatrixSize - 1): # if the updated Position[0] is outboard the matrix
 self.Position[0] = 0 # Move the female to the other side of the matrix
 elif DirectionPosition == 3: # if this object is equivalent to 3
 self.Position[1] += -1 # Move down the given female to one unit
 if self.Position[1] < 0: # if the updated Position[1] is outboard the matrix
 self.Position[1] = (GenParam.MatrixSize - 1) # Move the female along the top of the matrix
 elif DirectionPosition == 4: # if this object is equivalent to 4
 self.Position[1] += 1 # Move up the given female to one unit
 if self.Position[1] > (GenParam.MatrixSize - 1): # if the updated Position[0] is outboard the matrix
 self.Position[1] = 0 # Move the female to the other side of the matrix
 else:
 raise RuntimeError("error Direction position male")


 def IsAgentOverTerritory(self, TerritoryList):
 for TerritoryInstance in TerritoryList:
 if self.Position == TerritoryInstance.Position and TerritoryInstance.Availability == True:
 self.AgentOverTerritory = True
 self.AgentOverWhichIndexTerritory = TerritoryInstance.ID - 1
 #print("I am over a territory", self.IsAgentOverTerritory, " Patch nb",
 #self.AgentOverWhichIndexTerritory, "containing", TerritoryInstance.Quality, "food items",
 #"at", TerritoryInstance.Position)


 def ActionWhenAgentIsOverTerritory(self, TerritoryList):
 if TerritoryList[self.AgentOverWhichIndexTerritory].Quality > 0:
 if self.TimeBeforeAccessFood < self.AbilityToFindFood:
 self.TimeBeforeAccessFood += 1
 #print("i am the agent", self.ID, " i am wating :", self.TimeBeforeAccessFood, "before eat")
 else:
 TerritoryList[self.AgentOverWhichIndexTerritory].Quality -= 1
 #print(" patch nb :", TerritoryList[self.AgentOverWhichIndexTerritory].ID,
 # ", i have :", TerritoryList[self.AgentOverWhichIndexTerritory].Quality, "food item left")
 self.Quality += 1
 #print(" because the agent", self.ID, " ate", self.Quality, "food item")
 else:
 self.AgentOverTerritory = False
 #print("i am the agent", self.ID, "i am not eating anymore")
 #print("now i am here", self.Position, " i ate ", self.Quality, "food items")
 self.TimeBeforeAccessFood = 0
 Coordinate = 0
 CoordinateIsUnique = False
 while not (CoordinateIsUnique):
 CoordinateIsUnique = False
 Coordinate = [random.randint(0, GenParam.MatrixSize - 1),
 (random.randint(0, GenParam.MatrixSize - 1))]
 for TerritoryInstance in TerritoryList:
 if Coordinate == TerritoryInstance.Position:
 break
 else:
 CoordinateIsUnique = True
 TerritoryList[self.AgentOverWhichIndexTerritory].Position = Coordinate
 TerritoryList[self.AgentOverWhichIndexTerritory].Quality = GenParam.FoodPatchQuality
 # print("TerritoryID=", TerritoryInstance.ID, "containing", TerritoryInstance.Quality,
 # "food item, its localization is ", TerritoryInstance.Position,
 # "is it available?", TerritoryInstance.Availability)

# Class Female #

import random
import GeneralParameters as GenParam

class Female:
 def __init__(self, ID):
 self.ID = ID
 self.Position = []
 self.setInitialPosition()
 self.Precision = self.DefinePrecision()
 self.FoundMate = False
 self.AgentOverTerritory = False
 self.TerritoryVisited = []
 self.AgentOverWhichIndexTerritory = 0
 self.AgentOverWhichTerritoryID = 0
 self.TimeInFrontOfMate = 0
 self.EstimateMate = ()
 self.MateInitialCriterion = self.setMateInitialCriterion()
 self.Quality = 0
 self.FitnessCategory = 0
 self.ReproductiveSuccess = 0


 def __str__(self):
 return str(self.ID)+"\t\t"+\
 str(self.Precision)+"\t\t"+\
 str(self.MateInitialCriterion)+"\t\t"+\
 str(self.EstimateMate)+"\t\t"+\
 str(self.FoundMate)+"\t\t"+\
 str(self.Quality)+"\t\t"+\
 str(self.ReproductiveSuccess)+'\n'


 def setInitialPosition(self):
 self.Position = [random.randint(0, GenParam.MatrixSize - 1), random.randint(0, GenParam.MatrixSize - 1)]


 def AttributeMalePosition(self, TerritoryList):
 MaleIndex = random.randint(0, len(TerritoryList) - 1)
 self.Position = TerritoryList[MaleIndex].Position


 def DefinePrecision(self):
 Precision = random.randint(1, GenParam.TimeStepNbCompleteInformation)
 return Precision


 def setMateInitialCriterion(self):
 setMateInitialCriterion = random.randint(0, GenParam.MaleAbilityToFindFood)
 return setMateInitialCriterion


 def AttributionFitnessCategory(self, StatFitnessFemale):
 if self.Quality <=0:
 self.FitnessCategory = 0
 elif 1 <= self.Quality <= 10:
 self.FitnessCategory = 1
 elif 11 <= self.Quality <= 20:
 self.FitnessCategory = 2
 elif 21 <= self.Quality <= 30:
 self.FitnessCategory = 3
 elif 31 <= self.Quality <= 40:
 self.FitnessCategory = 4
 elif 41 <= self.Quality <= 50:
 self.FitnessCategory = 5
 elif 51 <= self.Quality <= 60:
 self.FitnessCategory = 6
 elif 61 <= self.Quality <= 70:
 self.FitnessCategory = 7
 elif 71 <= self.Quality <= 80:
 self.FitnessCategory = 8
 elif 81 <= self.Quality <= 90:
 self.FitnessCategory = 9
 elif 91 <= self.Quality <= 100:
 self.FitnessCategory = 10
 elif 101 <= self.Quality:
 self.FitnessCategory = 11
 else:
 raise RuntimeError("error fitness Category female")
 #print("femaleID", self.ID, "female fitness category", self.FitnessCategory)
 return self.FitnessCategory


 def EvolutionaryUpdateCharacteristic(self, ListHeritableTrait):
 IndexPrecision = random.randint(0, len(ListHeritableTrait[0])-1)
 #print("index precision", IndexPrecision)
 self.Precision = ListHeritableTrait[0].pop(IndexPrecision)
 #print("precision ListHeritableTrait", ListHeritableTrait[0], "Precision selected", self.Precision)
 IndexCriterion = random.randint(0, len(ListHeritableTrait[1])-1)
 #print("Index criterion", IndexCriterion)
 self.MateInitialCriterion = ListHeritableTrait[1].pop(IndexCriterion)
 #print("criterion ListHeritableTrait", ListHeritableTrait[1], "Criterion selected", self.MateInitialCriterion)
 self.setInitialPosition()
 self.FoundMate = False
 self.AgentOverTerritory = False
 self.TerritoryVisited = []
 self.AgentOverWhichIndexTerritory = 0
 self.AgentOverWhichTerritoryID = []
 self.TimeInFrontOfMate = 0
 self.EstimateMate = ()
 self.Quality = 0
 self.FitnessCategory = 0
 self.ReproductiveSuccess = 0


 def UpdatePosition(self, TerritoryList):
 MaleIndex = []
 #print("TerritoryVisited", self.TerritoryVisited)
 if self.TerritoryVisited != None:
 for TerritoryInstance in TerritoryList:
 if self.Position == TerritoryInstance.Position and TerritoryInstance.Availability == True:
 self.AgentOverWhichTerritoryID = TerritoryInstance.ID
 #print("AgentOverWhichTerritoryID", self.AgentOverWhichTerritoryID,
 # "TerritoryInstance.ID", TerritoryInstance.ID)
 for MateVisitID in self.TerritoryVisited:
 #print("TerritoryVisited list", self.TerritoryVisited, "MateVisitIndex", MateVisitID)
 if MateVisitID == self.AgentOverWhichTerritoryID and self.FoundMate == False:
 #print("MateVisitIndex",MateVisitID, "AgentOverWhichTerritoryID", self.AgentOverWhichTerritoryID)
 MaleIndex = random.randint(0, len(TerritoryList)-1)
 #print("MaleIndex", MaleIndex)
 self.Position = TerritoryList[MaleIndex].Position
 #print("localization of the female", self.Position,
 # "localization of the male", TerritoryList[MaleIndex].Position)


 def IsAgentOverTerritory(self, TerritoryList):
 for TerritoryInstance in TerritoryList:
 if self.Position == TerritoryInstance.Position and TerritoryInstance.Availability == True:
 self.AgentOverTerritory = True
 self.AgentOverWhichIndexTerritory = TerritoryInstance.ID - 1
 #print("I am over a territory", self.AgentOverTerritory, " Patch nb",
 #self.AgentOverWhichIndexTerritory, "containing", TerritoryInstance.Quality, "seeds",
 # "at", TerritoryInstance.Position)


### For simulation WITHOUT Sexual selection, use this function : females mate with the first male encounter
 #def ActionWhenAgentIsOverTerritory(self, TerritoryList): #
 # if self.AgentOverTerritory == True:
 # self.FoundMate = True
 # TerritoryList[self.AgentOverWhichIndexTerritory].Availability = False
 # #print("i found a mate :", self.FoundMate, " the male is not available anymore rigth?",
 # TerritoryList[self.AgentOverWhichIndexTerritory].Availability)
 # self.Quality = TerritoryList[self.AgentOverWhichIndexTerritory].Quality


### For simulation WITH Sexual selection, use this function : females prefer males with good cognitive abilities
 def ActionWhenAgentIsOverTerritory(self, TerritoryList): #
 if self.TimeInFrontOfMate < self.Precision and self.FoundMate == False:
 #print("i am the female nb", self.ID, "my precision = ", self.Precision,
 # "i am in front of a male since", self.TimeInFrontOfMate, "time step ")
 self.TimeInFrontOfMate += 1
 else:
 HeadsOrTails = random.randint(0, 1)

 if HeadsOrTails == 0:
 self.EstimateMate = TerritoryList[self.AgentOverWhichIndexTerritory].AbilityToFindFood -\
 (GenParam.TimeStepNbCompleteInformation - self.Precision)
 else:
 self.EstimateMate = TerritoryList[self.AgentOverWhichIndexTerritory].AbilityToFindFood +\
 (GenParam.TimeStepNbCompleteInformation - self.Precision)
 #print("i estimate him as = ", self.EstimateMate," 0 =- et 1 = + ", HeadsOrTails,
 # "with an error of:",ErrorEstimation,
 # "in reality his quality is ", TerritoryList[self.AgentOverWhichIndexTerritory].AbilityToFindFood)

 if self.EstimateMate <= self.MateInitialCriterion:
 self.FoundMate = True
 TerritoryList[self.AgentOverWhichIndexTerritory].Availability = False
 #print("i found a mate :", self.FoundMate,
 # "is the male still available?", TerritoryList[self.AgentOverWhichIndexTerritory].Availability)
 self.Quality = TerritoryList[self.AgentOverWhichIndexTerritory].Quality
 else:
 self.AgentOverTerritory = False
 self.TimeInFrontOfMate = 0
 self.TerritoryVisited.append(self.AgentOverWhichTerritoryID)
 self.EstimateMate = ()
 #print("TerritoryVisited", self.TerritoryVisited,
 # "AgentOverWhichTerritoryID", self.AgentOverWhichTerritoryID)

#### Main Data Analysis ####

import AnalyseData
import numpy


def main():
 #Scenario.SetScenarioNumber(GenParam.SelectedScenario)

 #Ouverture des fichiers outputs
 DisctributionMaleAbilityfile = open('DisctributionMaleAbilityfile.txt', 'w')
 DisctributionMaleAbilityfile.write("Range\t"
 "Population\t"
 "Generation\t"
 "Male\t"
 "AbilityToFindFood\n")

 DisctributionFemaleCriterionfile = open('DisctributionFemaleCriterionfile.txt', 'w')
 DisctributionFemaleCriterionfile.write("Range\t"
 "Population\t"
 "Generation\t"
 "Female\t"
 "Criterion\n")

 DisctributionFemalePrecisionfile = open('DisctributionFemalePrecisionfile.txt', 'w')
 DisctributionFemalePrecisionfile.write("Range\t"
 "Population\t"
 "Generation\t"
 "Female\t"
 "Precision\n")

 InterPopAnalyseParameterEffectfile = open('InterPopAnalyseParameterEffect.txt', 'w')
 InterPopAnalyseParameterEffectfile.write("Range\t"
 "InterPopulation50GenerationMeanAbility\t"
 "InterPopulation50GenerationSdAbility\t"
 "IntraPopulation50GenerationMeanSdAbility\t"
 "InterPopulation50GenerationMeanCriterion\t"
 "InterPopulation50GenerationSdCriterion\t"
 "IntraPopulation50GenerationMeanSdCriterion\t"
 "InterPopulation50GenerationMeanPrecision\t"
 "InterPopulation50GenerationSdPrecision\t"
 "IntraPopulation50GenerationMeanSdPrecision\n")

 IntraPopAnalyseParameterEffectfile = open('IntraPopAnalyseParameterEffect.txt', 'w')
 IntraPopAnalyseParameterEffectfile.write("Range\t"
 "Population\t"
 "IntraPopulation50GenerationMeanAbility\t"
 "IntraPopulation50GenerationSdAbility\t"
 "IntraPopulation50GenerationMeanCriterion\t"
 "IntraPopulation50GenerationSdCriterion\t"
 "IntraPopulation50GenerationMeanPrecision\t"
 "IntraPopulation50GenerationSdPrecision\n")

 IntraGenAnalyseParameterEffectfile = open('IntraGenAnalyseParameterEffect.txt', 'w')
 IntraGenAnalyseParameterEffectfile.write("Range\t"
 "Population\t"
 "Generation\t"
 "IntraGenerationMeanAbility\t"
 "IntraGenerationSdAbility\t"
 "IntraGenerationMeanCriterion\t"
 "IntraGenerationSdCriterion\t"
 "IntraGenerationMeanPrecision\t"
 "IntraGenerationSdPrecision\n")

 InterPopAnalyseParameterEffectBeginningfile = open('InterPopAnalyseParameterEffectBeginning.txt', 'w')
 InterPopAnalyseParameterEffectBeginningfile.write("Range\t"
 "InterPopulation50GenerationMeanAbility\t"
 "InterPopulation50GenerationSdAbility\t"
 "IntraPopulation50GenerationMeanSdAbility\t"
 "InterPopulation50GenerationMeanCriterion\t"
 "InterPopulation50GenerationSdCriterion\t"
 "IntraPopulation50GenerationMeanSdCriterion\t"
 "InterPopulation50GenerationMeanPrecision\t"
 "InterPopulation50GenerationSdPrecision\t"
 "IntraPopulation50GenerationMeanSdPrecision\n")

 IntraPopAnalyseParameterEffectBeginningfile = open('IntraPopAnalyseParameterEffectBeginning.txt', 'w')
 IntraPopAnalyseParameterEffectBeginningfile.write("Range\t"
 "Population\t"
 "IntraPopulation50GenerationMeanAbility\t"
 "IntraPopulation50GenerationSdAbility\t"
 "IntraPopulation50GenerationMeanCriterion\t"
 "IntraPopulation50GenerationSdCriterion\t"
 "IntraPopulation50GenerationMeanPrecision\t"
 "IntraPopulation50GenerationSdPrecision\n")

 IntraGenAnalyseParameterEffectBeginningfile = open('IntraGenAnalyseParameterEffectBeginning.txt', 'w')
 IntraGenAnalyseParameterEffectBeginningfile.write("Range\t"
 "Population\t"
 "Generation\t"
 "IntraGenerationMeanAbility\t"
 "IntraGenerationSdAbility\t"
 "IntraGenerationMeanCriterion\t"
 "IntraGenerationSdCriterion\t"
 "IntraGenerationMeanPrecision\t"
 "IntraGenerationSdPrecision\n")

 AnalyseParameterEffectfile = open('AnalyseParameterEffect.txt', 'w')
 AnalyseParameterEffectfile.write("Range\t"
 "MeanAbility\t"
 "SdAbility\t"
 "MeanCriterion\t"
 "SdCriterion\t"
 "MeanPrecision\t"
 "SdPrecision\n")

 AnalyseLinkFitnessMaleTraitEvolutionFile = open('AnalyseLinkFitnessMaleTraitEvolution.txt', 'w')
 AnalyseLinkFitnessMaleTraitEvolutionFile.write("Range\t"
 "Generation\t"
 "AbilityToFindFoodCategory\t"
 "MeanQuality\t"
 "SdQuality\n")

 AnalyseLinkFitnessFemaleCriterionEvolutionFile = open('AnalyseLinkFitnessFemaleCriterionEvolution.txt', 'w')
 AnalyseLinkFitnessFemaleCriterionEvolutionFile.write("Range\t"
 "Generation\t"
 "MateInitialCriterionCategory\t"
 "MeanQualitybyMateInitialCriterion\t"
 "SdQualitybyMateInitialCriterion\n")

 AnalyseLinkFitnessFemalePrecisionEvolutionFile = open('AnalyseLinkFitnessFemalePrecisionEvolution.txt', 'w')
 AnalyseLinkFitnessFemalePrecisionEvolutionFile.write("Range\t"
 "Generation\t"
 "PrecisionCategory\t"
 "MeanQualitybyPrecision\t"
 "SdQualitybyPrecision\n")

 AnalyseLinkSRMaleTraitEvolutionFile = open('AnalyseLinkSRMaleTraitEvolution.txt', 'w')
 AnalyseLinkSRMaleTraitEvolutionFile.write("Range\t"
 "Generation\t"
 "AbilityToFindFoodCategory\t"
 "MeanReprodSuccess\t"
 "SdReprodSuccess\n")

 AnalyseLinkSRFemaleCriterionEvolutionFile = open('AnalyseLinkSRFemaleCriterionEvolutionFile.txt', 'w')
 AnalyseLinkSRFemaleCriterionEvolutionFile.write("Range\t"
 "Generation\t"
 "MateInitialCriterionCategory\t"
 "MeanReprodSuccess\t"
 "SdReprodSuccess\n")

 AnalyseLinkSRFemalePrecisionEvolutionFile = open('AnalyseLinkSRFemalePrecisionEvolution.txt', 'w')
 AnalyseLinkSRFemalePrecisionEvolutionFile.write("Range\t"
 "Generation\t"
 "PrecisionCategory\t"
 "MeanReprodSuccess\t"
 "SdReprodSuccess\n")


 #Calculate and save mean trait values across all ranges of the parameters
 with open('DATABRUTEAbilityValueByRangeByRepetitionByGeneration.npy', 'rb') as f:
 AbilityValueByRangeByRepetitionByGeneration = numpy.load(f, allow_pickle=True)
 with open('DATABRUTEMateChoiceCriterionValueByRangeByRepetitionByGeneration.npy', 'rb') as f:
 MateChoiceCriterionValueByRangeByRepetitionByGeneration = numpy.load(f, allow_pickle=True)
 with open('DATABRUTEPrecisionValueByRangeByRepetitionByGeneration.npy', 'rb') as f:
 PrecisionValueByRangeByRepetitionByGeneration = numpy.load(f, allow_pickle=True)
 with open('DATABRUTEFitnessByRangeByRepetitionByGenerationByAbility.npy', 'rb') as f:
 FitnessByRangeByRepetitionByGenerationByAbility = numpy.load(f, allow_pickle=True)
 with open('DATABRUTEFitnessByRangeByRepetitionByGenerationByMateChoiceCriterion.npy', 'rb') as f:
 FitnessByRangeByRepetitionByGenerationByMateChoiceCriterion = numpy.load(f, allow_pickle=True)
 with open('DATABRUTEFitnessByRangeByRepetitionByGenerationByPrecision.npy', 'rb') as f:
 FitnessByRangeByRepetitionByGenerationByPrecision = numpy.load(f, allow_pickle=True)
 with open('DATABRUTEReprodSuccessByRangeByRepetitionByGenerationByAbility.npy', 'rb') as f:
 ReprodSuccessByRangeByRepetitionByGenerationByAbility = numpy.load(f, allow_pickle=True)
 with open('DATABRUTEReprodSuccessByRangeByRepetitionByGenerationByMateChoiceCriterion.npy', 'rb') as f:
 ReprodSuccessByRangeByRepetitionByGenerationByMateChoiceCriterion = numpy.load(f, allow_pickle=True)
 with open('DATABRUTEReprodSuccessByRangeByRepetitionByGenerationByPrecision.npy', 'rb') as f:
 ReprodSuccessByRangeByRepetitionByGenerationByPrecision = numpy.load(f, allow_pickle=True)

 # Calculate and save the final data
 AnalyseData.AnalyseParameterEffect(AbilityValueByRangeByRepetitionByGeneration.tolist(),
 MateChoiceCriterionValueByRangeByRepetitionByGeneration.tolist(),
 PrecisionValueByRangeByRepetitionByGeneration.tolist(),
 IntraGenAnalyseParameterEffectfile,
 IntraPopAnalyseParameterEffectfile,
 InterPopAnalyseParameterEffectfile)
 print("bla1")

 AnalyseData.AnalyseParameterEffectBeginning(AbilityValueByRangeByRepetitionByGeneration.tolist(),
 MateChoiceCriterionValueByRangeByRepetitionByGeneration.tolist(),
 PrecisionValueByRangeByRepetitionByGeneration.tolist(),
 IntraGenAnalyseParameterEffectBeginningfile,
 IntraPopAnalyseParameterEffectBeginningfile,
 InterPopAnalyseParameterEffectBeginningfile)
 print("bla2")

 AnalyseData.MeanFitnessMaleByAbility(FitnessByRangeByRepetitionByGenerationByAbility.tolist(),
 AnalyseLinkFitnessMaleTraitEvolutionFile)
 print("bla3")

 AnalyseData.AnalyseLinkFitnessFemaleCriterion(FitnessByRangeByRepetitionByGenerationByMateChoiceCriterion.tolist(),
 AnalyseLinkFitnessFemaleCriterionEvolutionFile)
 AnalyseData.AnalyseLinkFitnessFemalePrecision(FitnessByRangeByRepetitionByGenerationByPrecision.tolist(),
 AnalyseLinkFitnessFemalePrecisionEvolutionFile)
 AnalyseData.AnalyseLinkReprodSuccessMaleTrait(ReprodSuccessByRangeByRepetitionByGenerationByAbility.tolist(),
 AnalyseLinkSRMaleTraitEvolutionFile)
 AnalyseData.AnalyseLinkReprodSuccessFemaleCriterion(ReprodSuccessByRangeByRepetitionByGenerationByMateChoiceCriterion.tolist(),
 AnalyseLinkSRFemaleCriterionEvolutionFile)
 print("bla4")

 AnalyseData.AnalyseLinkReprodSuccessFemalePrecision(ReprodSuccessByRangeByRepetitionByGenerationByPrecision.tolist(),
 AnalyseLinkSRFemalePrecisionEvolutionFile)
 AnalyseData.DistributionMaleAbility(AbilityValueByRangeByRepetitionByGeneration.tolist(),
 DisctributionMaleAbilityfile)
 print("bla5")

 AnalyseData.DistributionFemaleCriterion(MateChoiceCriterionValueByRangeByRepetitionByGeneration.tolist(),
 DisctributionFemaleCriterionfile)
 print("bla6")

 AnalyseData.DistributionFemalePrecision(PrecisionValueByRangeByRepetitionByGeneration.tolist(),
 DisctributionFemalePrecisionfile)


if __name__ == '__main__':
 main()

### Data Analysis ###

import GeneralParameters as GenParam
import math

def DistributionMaleAbility(
 AbilityValueByRangeByRepetitionByGeneration,
 DisctributionMaleAbilityfile):
 for sc in range(GenParam.Range.value):
 for r in range(GenParam.TotalNumberOfScenarioRepetition):
 for s in range(GenParam.TotalNumberOfSimulation):
 for MaleIndex in range(GenParam.MaleNumber):
 Ability = AbilityValueByRangeByRepetitionByGeneration[sc][r][s][0][MaleIndex]
 DisctributionMaleAbilityfile.write(str(sc) + "\t" +
 str(r) + "\t" +
 str(s) + "\t" +
 str(MaleIndex) + "\t" +
 str(Ability) +"\n")

def DistributionFemaleCriterion(
 MateChoiceCriterionValueByRangeByRepetitionByGeneration,
 DisctributionFemaleCriterionfile):
 for sc in range(GenParam.Range.value):
 for r in range(GenParam.TotalNumberOfScenarioRepetition):
 for s in range(GenParam.TotalNumberOfSimulation):
 for FemaleIndex in range(GenParam.FemaleNumber):
 Criterion = MateChoiceCriterionValueByRangeByRepetitionByGeneration[sc][r][s][0][FemaleIndex]
 DisctributionFemaleCriterionfile.write(str(sc) + "\t" +
 str(r) + "\t" +
 str(s) + "\t" +
 str(FemaleIndex) + "\t" +
 str(Criterion) +"\n")

def DistributionFemalePrecision(
 PrecisionValueByRangeByRepetitionByGeneration,
 DisctributionFemalePrecisionfile):
 for sc in range(GenParam.Range.value):
 for r in range(GenParam.TotalNumberOfScenarioRepetition):
 for s in range(GenParam.TotalNumberOfSimulation):
 for FemaleIndex in range(GenParam.FemaleNumber):
 Precision = PrecisionValueByRangeByRepetitionByGeneration[sc][r][s][0][FemaleIndex]
 DisctributionFemalePrecisionfile.write(str(sc) + "\t" +
 str(r) + "\t" +
 str(s) + "\t" +
 str(FemaleIndex) + "\t" +
 str(Precision) +"\n")

def AnalyseParameterEffect(
 AbilityValueByRangeByRepetitionByGeneration,
 MateChoiceCriterionValueByRangeByRepetitionByGeneration,
 PrecisionValueByRangeByRepetitionByGeneration,
 IntraGenAnalyseParameterEffectfile,
 IntraPopAnalyseParameterEffectfile,
 InterPopAnalyseParameterEffectfile):
 for sc in range(GenParam.Range.value):
 IntraPopulation50GenerationMeanAbilityList = []
 IntraPopulation50GenerationSdAbilityList = []
 IntraPopulation50GenerationMeanCriterionList = []
 IntraPopulation50GenerationSdCriterionList = []
 IntraPopulation50GenerationPrecisionList = []
 IntraPopulation50GenerationSdPrecisionList = []
 for r in range(GenParam.TotalNumberOfScenarioRepetition):
 IntraGenerationMeanAbilityList = []
 IntraGenerationSdAbilityList = []
 IntraGenerationMeanCriterionList = []
 IntraGenerationSdCriterionList = []
 IntraGenerationMeanPrecisionList = []
 IntraGenerationSdPrecisionList = []
 for s in range((GenParam.TotalNumberOfSimulation-50), GenParam.TotalNumberOfSimulation):
 AbilityList = AbilityValueByRangeByRepetitionByGeneration[sc][r][s][0]
 #print(AbilityList)
 IntraGenerationSumAbility = sum(AbilityList)
 # print(len(AbilityValueByRangeByRepetitionByGeneration[sc][r][s]))
 IntraGenerationMeanAbility = IntraGenerationSumAbility / len(AbilityList)
 IntraGenerationMeanAbilityList.append(IntraGenerationMeanAbility)
 IntraGenerationSdAbility = math.sqrt(sum([((x - IntraGenerationMeanAbility) ** 2)
 for x in AbilityList]) / len(AbilityList))
 IntraGenerationSdAbilityList.append(IntraGenerationSdAbility)

 CriterionList = MateChoiceCriterionValueByRangeByRepetitionByGeneration[sc][r][s][0]
 IntraGenerationSumCriterion = sum(CriterionList)
 # print(len(MateChoiceCriterionValueByRangeByRepetitionByGeneration[sc][r][s]))
 IntraGenerationMeanCriterion = IntraGenerationSumCriterion / len(CriterionList)
 IntraGenerationMeanCriterionList.append(IntraGenerationMeanCriterion)
 IntraGenerationSdCriterion = math.sqrt(sum([((x - IntraGenerationMeanCriterion) ** 2)
 for x in CriterionList]) / len(CriterionList))
 IntraGenerationSdCriterionList.append(IntraGenerationSdCriterion)

 PrecisionList = PrecisionValueByRangeByRepetitionByGeneration[sc][r][s][0]
 IntraGenerationSumPrecision = sum(PrecisionList)
 # print(len(PrecisionValueByRangeByRepetitionByGeneration[sc][r][s]))
 IntraGenerationMeanPrecision = IntraGenerationSumPrecision / len(PrecisionList)
 IntraGenerationMeanPrecisionList.append(IntraGenerationMeanPrecision)
 IntraGenerationSdPrecision = math.sqrt(sum([((x - IntraGenerationMeanPrecision) ** 2)
 for x in PrecisionList]) / len(PrecisionList))
 IntraGenerationSdPrecisionList.append(IntraGenerationSdPrecision)
 IntraGenAnalyseParameterEffectfile.write(str(sc) + "\t" +
 str(r) + "\t" +
 str(s) + "\t" +
 str(IntraGenerationMeanAbility) + "\t" +
 str(IntraGenerationSdAbility) + "\t" +
 str(IntraGenerationMeanCriterion) + "\t" +
 str(IntraGenerationSdCriterion) + "\t" +
 str(IntraGenerationMeanPrecision) + "\t" +
 str(IntraGenerationSdPrecision) + "\n")

 IntraPopulation50GenerationMeanAbility = sum(IntraGenerationMeanAbilityList) / \
 len(IntraGenerationMeanAbilityList)
 IntraPopulation50GenerationMeanAbilityList.append(IntraPopulation50GenerationMeanAbility)
 IntraPopulation50GenerationSdAbility = sum(IntraGenerationSdAbilityList) / \
 len(IntraGenerationSdAbilityList)
 IntraPopulation50GenerationSdAbilityList.append(IntraPopulation50GenerationSdAbility)

 IntraPopulation50GenerationMeanCriterion = sum(IntraGenerationMeanCriterionList) / \
 len(IntraGenerationMeanCriterionList)
 IntraPopulation50GenerationMeanCriterionList.append(IntraPopulation50GenerationMeanCriterion)
 IntraPopulation50GenerationSdCriterion = sum(IntraGenerationSdCriterionList) / \
 len(IntraGenerationSdCriterionList)
 IntraPopulation50GenerationSdCriterionList.append(IntraPopulation50GenerationSdCriterion)

 IntraPopulation50GenerationPrecision = sum(IntraGenerationMeanPrecisionList) / \
 len(IntraGenerationMeanPrecisionList)
 IntraPopulation50GenerationPrecisionList.append(IntraPopulation50GenerationPrecision)
 IntraPopulation50GenerationSdPrecision = sum(IntraGenerationSdPrecisionList) / \
 len(IntraGenerationSdPrecisionList)
 IntraPopulation50GenerationSdPrecisionList.append(IntraPopulation50GenerationSdPrecision)

 IntraPopAnalyseParameterEffectfile.write(str(sc) + "\t" +
 str(r) + "\t" +
 str(IntraPopulation50GenerationMeanAbility) + "\t" +
 str(IntraPopulation50GenerationSdAbility) + "\t" +
 str(IntraPopulation50GenerationMeanCriterion) + "\t" +
 str(IntraPopulation50GenerationSdCriterion) + "\t" +
 str(IntraPopulation50GenerationPrecision) + "\t" +
 str(IntraPopulation50GenerationSdPrecision) + "\n")

 InterPopulation50GenerationMeanAbility = sum(IntraPopulation50GenerationMeanAbilityList) / \
 len(IntraPopulation50GenerationMeanAbilityList)
 InterPopulation50GenerationSdAbility = math.sqrt(sum([((x - InterPopulation50GenerationMeanAbility) ** 2)
 for x in IntraPopulation50GenerationMeanAbilityList]) /
 len(IntraPopulation50GenerationMeanAbilityList))
 IntraPopulation50GenerationMeanSdAbility = sum(IntraPopulation50GenerationSdAbilityList) / \
 len(IntraPopulation50GenerationSdAbilityList)

 InterPopulation50GenerationMeanCriterion = sum(IntraPopulation50GenerationMeanCriterionList) / \
 len(IntraPopulation50GenerationMeanCriterionList)
 InterPopulation50GenerationSdCriterion = math.sqrt(sum([((x - InterPopulation50GenerationMeanCriterion) ** 2)
 for x in IntraPopulation50GenerationMeanCriterionList])/
 len(IntraPopulation50GenerationMeanCriterionList))
 IntraPopulation50GenerationMeanSdCriterion = sum(IntraPopulation50GenerationSdCriterionList) / \
 len(IntraPopulation50GenerationSdCriterionList)

 InterPopulation50GenerationMeanPrecision = sum(IntraPopulation50GenerationPrecisionList) / \
 len(IntraPopulation50GenerationPrecisionList)
 InterPopulation50GenerationSdPrecision = math.sqrt(sum([((x - InterPopulation50GenerationMeanPrecision) ** 2)
 for x in IntraPopulation50GenerationPrecisionList]) /
 len(IntraPopulation50GenerationPrecisionList))
 IntraPopulation50GenerationMeanSdPrecision = sum(IntraPopulation50GenerationSdPrecisionList) / \
 len(IntraPopulation50GenerationSdPrecisionList)

 InterPopAnalyseParameterEffectfile.write(str(sc) + "\t" +
 str(InterPopulation50GenerationMeanAbility) + "\t" +
 str(InterPopulation50GenerationSdAbility) + "\t" +
 str(IntraPopulation50GenerationMeanSdAbility) + "\t" +
 str(InterPopulation50GenerationMeanCriterion) + "\t" +
 str(InterPopulation50GenerationSdCriterion) + "\t" +
 str(IntraPopulation50GenerationMeanSdCriterion) + "\t" +
 str(InterPopulation50GenerationMeanPrecision) + "\t" +
 str(InterPopulation50GenerationSdPrecision) + "\t" +
 str(IntraPopulation50GenerationMeanSdPrecision) + "\n")

 IntraGenAnalyseParameterEffectfile.close()
 IntraPopAnalyseParameterEffectfile.close()
 InterPopAnalyseParameterEffectfile.close()

def AnalyseParameterEffectBeginning(
 AbilityValueByRangeByRepetitionByGeneration,
 MateChoiceCriterionValueByRangeByRepetitionByGeneration,
 PrecisionValueByRangeByRepetitionByGeneration,
 IntraGenAnalyseParameterEffectBeginningfile,
 IntraPopAnalyseParameterEffectBeginningfile,
 InterPopAnalyseParameterEffectBeginningfile):
 for sc in range(GenParam.Range.value):
 IntraPopulation50GenerationMeanAbilityList = []
 IntraPopulation50GenerationSdAbilityList = []
 IntraPopulation50GenerationMeanCriterionList = []
 IntraPopulation50GenerationSdCriterionList = []
 IntraPopulation50GenerationPrecisionList = []
 IntraPopulation50GenerationSdPrecisionList = []
 for r in range(GenParam.TotalNumberOfScenarioRepetition):
 IntraGenerationMeanAbilityList = []
 IntraGenerationSdAbilityList = []
 IntraGenerationMeanCriterionList = []
 IntraGenerationSdCriterionList = []
 IntraGenerationMeanPrecisionList = []
 IntraGenerationSdPrecisionList = []
 for s in range(50):
 AbilityList = AbilityValueByRangeByRepetitionByGeneration[sc][r][s][0]
 # print(AbilityList)
 IntraGenerationSumAbility = sum(AbilityList)
 # print(len(AbilityValueByRangeByRepetitionByGeneration[sc][r][s]))
 IntraGenerationMeanAbility = IntraGenerationSumAbility / len(AbilityList)
 IntraGenerationMeanAbilityList.append(IntraGenerationMeanAbility)
 IntraGenerationSdAbility = math.sqrt(sum([((x - IntraGenerationMeanAbility) ** 2)
 for x in AbilityList]) / len(AbilityList))
 IntraGenerationSdAbilityList.append(IntraGenerationSdAbility)

 CriterionList = MateChoiceCriterionValueByRangeByRepetitionByGeneration[sc][r][s][0]
 IntraGenerationSumCriterion = sum(CriterionList)
 # print(len(MateChoiceCriterionValueByRangeByRepetitionByGeneration[sc][r][s]))
 IntraGenerationMeanCriterion = IntraGenerationSumCriterion / len(CriterionList)
 IntraGenerationMeanCriterionList.append(IntraGenerationMeanCriterion)
 IntraGenerationSdCriterion = math.sqrt(sum([((x - IntraGenerationMeanCriterion) ** 2)
 for x in CriterionList]) / len(CriterionList))
 IntraGenerationSdCriterionList.append(IntraGenerationSdCriterion)

 PrecisionList = PrecisionValueByRangeByRepetitionByGeneration[sc][r][s][0]
 IntraGenerationSumPrecision = sum(PrecisionList)
 # print(len(PrecisionValueByRangeByRepetitionByGeneration[sc][r][s]))
 IntraGenerationMeanPrecision = IntraGenerationSumPrecision / len(PrecisionList)
 IntraGenerationMeanPrecisionList.append(IntraGenerationMeanPrecision)
 IntraGenerationSdPrecision = math.sqrt(sum([((x - IntraGenerationMeanPrecision) ** 2)
 for x in PrecisionList]) / len(PrecisionList))
 IntraGenerationSdPrecisionList.append(IntraGenerationSdPrecision)

 IntraGenAnalyseParameterEffectBeginningfile.write(str(sc) + "\t" +
 str(r) + "\t" +
 str(s) + "\t" +
 str(IntraGenerationMeanAbility) + "\t" +
 str(IntraGenerationSdAbility) + "\t" +
 str(IntraGenerationMeanCriterion) + "\t" +
 str(IntraGenerationSdCriterion) + "\t" +
 str(IntraGenerationMeanPrecision) + "\t" +
 str(IntraGenerationSdPrecision) + "\n")

 IntraPopulation50GenerationMeanAbility = sum(IntraGenerationMeanAbilityList) / \
 len(IntraGenerationMeanAbilityList)
 IntraPopulation50GenerationMeanAbilityList.append(IntraPopulation50GenerationMeanAbility)
 IntraPopulation50GenerationSdAbility = sum(IntraGenerationSdAbilityList) / \
 len(IntraGenerationSdAbilityList)
 IntraPopulation50GenerationSdAbilityList.append(IntraPopulation50GenerationSdAbility)
 IntraPopulation50GenerationMeanCriterion = sum(IntraGenerationMeanCriterionList) / \
 len(IntraGenerationMeanCriterionList)
 IntraPopulation50GenerationMeanCriterionList.append(IntraPopulation50GenerationMeanCriterion)
 IntraPopulation50GenerationSdCriterion = sum(IntraGenerationSdCriterionList) / \
 len(IntraGenerationSdCriterionList)
 IntraPopulation50GenerationSdCriterionList.append(IntraPopulation50GenerationSdCriterion)
 IntraPopulation50GenerationPrecision = sum(IntraGenerationMeanPrecisionList) /\
 len(IntraGenerationMeanPrecisionList)
 IntraPopulation50GenerationPrecisionList.append(IntraPopulation50GenerationPrecision)
 IntraPopulation50GenerationSdPrecision = sum(IntraGenerationSdPrecisionList) / \
 len(IntraGenerationSdPrecisionList)
 IntraPopulation50GenerationSdPrecisionList.append(IntraPopulation50GenerationSdPrecision)

 IntraPopAnalyseParameterEffectBeginningfile.write(str(sc) + "\t" +
 str(r) + "\t" +
 str(IntraPopulation50GenerationMeanAbility) + "\t" +
 str(IntraPopulation50GenerationSdAbility) + "\t" +
 str(IntraPopulation50GenerationMeanCriterion) + "\t" +
 str(IntraPopulation50GenerationSdCriterion) + "\t" +
 str(IntraPopulation50GenerationPrecision) + "\t" +
 str(IntraPopulation50GenerationSdPrecision) + "\n")

 InterPopulation50GenerationMeanAbility = sum(IntraPopulation50GenerationMeanAbilityList) / \
 len(IntraPopulation50GenerationMeanAbilityList)
 InterPopulation50GenerationSdAbility = math.sqrt(sum([((x - InterPopulation50GenerationMeanAbility) ** 2)
 for x in IntraPopulation50GenerationMeanAbilityList]) /
 len(IntraPopulation50GenerationMeanAbilityList))
 IntraPopulation50GenerationMeanSdAbility = sum(IntraPopulation50GenerationSdAbilityList) / \
 len(IntraPopulation50GenerationSdAbilityList)
 InterPopulation50GenerationMeanCriterion = sum(IntraPopulation50GenerationMeanCriterionList) / \
 len(IntraPopulation50GenerationMeanCriterionList)
 InterPopulation50GenerationSdCriterion = math.sqrt(sum([((x - InterPopulation50GenerationMeanCriterion) ** 2)
 for x in IntraPopulation50GenerationMeanCriterionList])
 / len(IntraPopulation50GenerationMeanCriterionList))
 IntraPopulation50GenerationMeanSdCriterion = sum(IntraPopulation50GenerationSdCriterionList) / \
 len(IntraPopulation50GenerationSdCriterionList)
 InterPopulation50GenerationMeanPrecision = sum(IntraPopulation50GenerationPrecisionList) / \
 len(IntraPopulation50GenerationPrecisionList)
 InterPopulation50GenerationSdPrecision = math.sqrt(sum([((x - InterPopulation50GenerationMeanPrecision) ** 2)
 for x in IntraPopulation50GenerationPrecisionList]) /
 len(IntraPopulation50GenerationPrecisionList))
 IntraPopulation50GenerationMeanSdPrecision = sum(IntraPopulation50GenerationSdPrecisionList) / \
 len(IntraPopulation50GenerationSdPrecisionList)

 InterPopAnalyseParameterEffectBeginningfile.write(str(sc) + "\t" +
 str(InterPopulation50GenerationMeanAbility) + "\t" +
 str(InterPopulation50GenerationSdAbility) + "\t" +
 str(IntraPopulation50GenerationMeanSdAbility) + "\t" +
 str(InterPopulation50GenerationMeanCriterion) + "\t" +
 str(InterPopulation50GenerationSdCriterion) + "\t" +
 str(IntraPopulation50GenerationMeanSdCriterion) + "\t" +
 str(InterPopulation50GenerationMeanPrecision) + "\t" +
 str(InterPopulation50GenerationSdPrecision) + "\t" +
 str(IntraPopulation50GenerationMeanSdPrecision) + "\n")

 IntraGenAnalyseParameterEffectBeginningfile.close()
 IntraPopAnalyseParameterEffectBeginningfile.close()
 InterPopAnalyseParameterEffectBeginningfile.close()

def MeanFitnessMaleByAbility(
 FitnessByRangeByRepetitionByGenerationByAbility,
 AnalyseLinkFitnessMaleTraitEvolutionFile):
 MeanSDQualityOverRepetition = []
 for sc in range(GenParam.Range.value):
 MeanSDQualityOverRepetition.append([])
 for s in range(GenParam.TotalNumberOfSimulation):
 MeanSDQualityOverRepetition[sc].append([])
 for r in range(GenParam.TotalNumberOfScenarioRepetition):
 MeanSDQualityOverRepetition[sc][s].append([])
 for AbilityIndex in range(GenParam.MaleAbilityToFindFood+1):
 MeanSDQualityOverRepetition[sc][s][r].append([])
 for sc in range(GenParam.Range.value):
 for s in range(GenParam.TotalNumberOfSimulation):
 for r in range(GenParam.TotalNumberOfScenarioRepetition):
 MeanList = []
 SdList = []
 for AbilityIndex in range(GenParam.MaleAbilityToFindFood+1):
 if len(FitnessByRangeByRepetitionByGenerationByAbility[sc][r][s][0][AbilityIndex]) != 0:
 SumQuality = sum(FitnessByRangeByRepetitionByGenerationByAbility[sc][r][s][0][AbilityIndex])
 #print(len(FitnessByRangeByRepetitionByGenerationByAbility[sc][r][s][0][AbilityIndex]))
 MeanQuality = SumQuality/\
 len(FitnessByRangeByRepetitionByGenerationByAbility[sc][r][s][0][AbilityIndex])
 MeanList.append(MeanQuality)
 SdQuality = math.sqrt(sum([((x - MeanQuality) ** 2) for x in FitnessByRangeByRepetitionByGenerationByAbility[sc][r][s][0][AbilityIndex]]) / len(FitnessByRangeByRepetitionByGenerationByAbility[sc][r][s][0][AbilityIndex]))
 SdList.append(SdQuality)
 MeanSDQualityOverRepetition[sc][s][r][AbilityIndex].append(MeanQuality)
 MeanSDQualityOverRepetition[sc][s][r][AbilityIndex].append(SdQuality)
 #print(MeanSDQualityOverRepetition[sc][s][r][AbilityIndex])
 else:
 MeanSDQualityOverRepetition[sc][s][r][AbilityIndex].append(0)
 MeanSDQualityOverRepetition[sc][s][r][AbilityIndex].append(0)
 #print(MeanSDQualityOverRepetition[sc][s][r][AbilityIndex])
 for sc in range(GenParam.Range.value):
 for s in range(GenParam.TotalNumberOfSimulation):
 MeanList = []
 SdList = []
 for AbilityIndex in range(GenParam.MaleAbilityToFindFood+1):
 SumMeanQuality = 0
 SumSDQuality = 0
 for r in range(GenParam.TotalNumberOfScenarioRepetition):
 SumMeanQuality = SumMeanQuality + MeanSDQualityOverRepetition[sc][s][r][AbilityIndex][0]
 SumSDQuality = SumSDQuality + MeanSDQualityOverRepetition[sc][s][r][AbilityIndex][1]
 #print(SumMeanQuality)
 #print(SumSDQuality)
 MeanMeanQuality = SumMeanQuality / (GenParam.TotalNumberOfScenarioRepetition)
 MeanList.append(MeanMeanQuality)
 MeanSdQuality = SumSDQuality / (GenParam.TotalNumberOfScenarioRepetition)
 SdList.append(MeanSdQuality)
 AnalyseLinkFitnessMaleTraitEvolutionFile.write(str(sc) + "\t" +
 str(s) + "\t" +
 str(AbilityIndex) + "\t" +
 str(MeanMeanQuality) + "\t" +
 str(MeanSdQuality) + "\n")

 AnalyseLinkFitnessMaleTraitEvolutionFile.close()

def AnalyseLinkFitnessFemaleCriterion(
 FitnessByRangeByRepetitionByGenerationByMateChoiceCriterion,
 AnalyseLinkFitnessFemaleCriterionEvolutionFile):
 MeanSDCriterionOverRepetition = []
 for sc in range(GenParam.Range.value):
 MeanSDCriterionOverRepetition.append([])
 for s in range(GenParam.TotalNumberOfSimulation):
 MeanSDCriterionOverRepetition[sc].append([])
 for r in range(GenParam.TotalNumberOfScenarioRepetition):
 MeanSDCriterionOverRepetition[sc][s].append([])
 for CriterionIndex in range(GenParam.MaleAbilityToFindFood+1):
 MeanSDCriterionOverRepetition[sc][s][r].append([])
 for sc in range(GenParam.Range.value):
 for s in range(GenParam.TotalNumberOfSimulation):
 for r in range(GenParam.TotalNumberOfScenarioRepetition):
 MeanList = []
 SdList = []
 for CriterionIndex in range(GenParam.MaleAbilityToFindFood+1):
 if len(FitnessByRangeByRepetitionByGenerationByMateChoiceCriterion[sc][r][s][0][CriterionIndex]) != 0:
 SumQuality = sum(FitnessByRangeByRepetitionByGenerationByMateChoiceCriterion[sc][r][s][0][CriterionIndex])
 #print(len(FitnessByRangeByRepetitionByGenerationByMateChoiceCriterion[sc][r][s][0][AbilityIndex]))
 MeanQuality = SumQuality/len(FitnessByRangeByRepetitionByGenerationByMateChoiceCriterion[sc][r][s][0][CriterionIndex])
 #MeanList.append(MeanQuality)
 SdQuality = math.sqrt(sum([((x - MeanQuality) ** 2) for x in FitnessByRangeByRepetitionByGenerationByMateChoiceCriterion[sc][r][s][0][CriterionIndex]]) / len(FitnessByRangeByRepetitionByGenerationByMateChoiceCriterion[sc][r][s][0][CriterionIndex]))
 #SdList.append(SdQuality)
 MeanSDCriterionOverRepetition[sc][s][r][CriterionIndex].append(MeanQuality)
 MeanSDCriterionOverRepetition[sc][s][r][CriterionIndex].append(SdQuality)
 #print(MeanSDCriterionOverRepetition[sc][s][r][CriterionIndex])

 else:
 MeanSDCriterionOverRepetition[sc][s][r][CriterionIndex].append(0)
 MeanSDCriterionOverRepetition[sc][s][r][CriterionIndex].append(0)
 #print(MeanSDCriterionOverRepetition[sc][s][r][CriterionIndex])
 for sc in range(GenParam.Range.value):
 for s in range(GenParam.TotalNumberOfSimulation):
 MeanList = []
 SdList = []
 for CriterionIndex in range(GenParam.MaleAbilityToFindFood+1):
 SumMeanQuality = 0
 SumSDQuality = 0
 for r in range(GenParam.TotalNumberOfScenarioRepetition):
 SumMeanQuality = SumMeanQuality + MeanSDCriterionOverRepetition[sc][s][r][CriterionIndex][0]
 SumSDQuality = SumSDQuality + MeanSDCriterionOverRepetition[sc][s][r][CriterionIndex][1]
 #print(SumMeanQuality)
 #print(SumSDQuality)
 MeanMeanQuality = SumMeanQuality / (GenParam.TotalNumberOfScenarioRepetition)
 MeanList.append(MeanMeanQuality)
 MeanSdQuality = SumSDQuality / (GenParam.TotalNumberOfScenarioRepetition)
 SdList.append(MeanSdQuality)
 AnalyseLinkFitnessFemaleCriterionEvolutionFile.write(str(sc) + "\t" +
 str(s) + "\t" +
 str(CriterionIndex) + "\t" +
 str(MeanMeanQuality) + "\t" +
 str(MeanSdQuality) + "\n")
 AnalyseLinkFitnessFemaleCriterionEvolutionFile.close()

def AnalyseLinkFitnessFemalePrecision(
 FitnessByRangeByRepetitionByGenerationByPrecision,
 AnalyseLinkFitnessFemalePrecisionEvolutionFile):
 MeanSDPrecisionOverRepetition = []
 for sc in range(GenParam.Range.value):
 MeanSDPrecisionOverRepetition.append([])
 for s in range(GenParam.TotalNumberOfSimulation):
 MeanSDPrecisionOverRepetition[sc].append([])
 for r in range(GenParam.TotalNumberOfScenarioRepetition):
 MeanSDPrecisionOverRepetition[sc][s].append([])
 for PrecisionIndex in range(GenParam.TimeStepNbCompleteInformation+1):
 MeanSDPrecisionOverRepetition[sc][s][r].append([])
 for sc in range(GenParam.Range.value):
 for s in range(GenParam.TotalNumberOfSimulation):
 for r in range(GenParam.TotalNumberOfScenarioRepetition):
 MeanList = []
 SdList = []
 for PrecisionIndex in range(GenParam.TimeStepNbCompleteInformation+1):
 if len(FitnessByRangeByRepetitionByGenerationByPrecision[sc][r][s][0][PrecisionIndex]) != 0:
 SumQuality = sum(FitnessByRangeByRepetitionByGenerationByPrecision[sc][r][s][0][PrecisionIndex])
 #print(len(FitnessByRangeByRepetitionByGenerationByPrecision[sc][0][s][0][PrecisionIndex]))
 MeanQuality = SumQuality/len(FitnessByRangeByRepetitionByGenerationByPrecision[sc][r][s][0][PrecisionIndex])
 #MeanList.append(MeanQuality)
 SdQuality = math.sqrt(sum([((x - MeanQuality) ** 2) for x in FitnessByRangeByRepetitionByGenerationByPrecision[sc][r][s][0][PrecisionIndex]]) / len(FitnessByRangeByRepetitionByGenerationByPrecision[sc][r][s][0][PrecisionIndex]))
 #SdList.append(SdQuality)
 MeanSDPrecisionOverRepetition[sc][s][r][PrecisionIndex].append(MeanQuality)
 MeanSDPrecisionOverRepetition[sc][s][r][PrecisionIndex].append(SdQuality)
 #print(MeanSDPrecisionOverRepetition[sc][s][r][PrecisionIndex])
 else:
 MeanSDPrecisionOverRepetition[sc][s][r][PrecisionIndex].append(0)
 MeanSDPrecisionOverRepetition[sc][s][r][PrecisionIndex].append(0)
 #print(MeanSDPrecisionOverRepetition[sc][s][r][PrecisionIndex])
 for sc in range(GenParam.Range.value):
 for s in range(GenParam.TotalNumberOfSimulation):
 MeanList = []
 SdList = []
 for PrecisionIndex in range(1, GenParam.TimeStepNbCompleteInformation +1):
 SumMeanQuality = 0
 SumSDQuality = 0
 for r in range(GenParam.TotalNumberOfScenarioRepetition):
 SumMeanQuality = SumMeanQuality + MeanSDPrecisionOverRepetition[sc][s][r][PrecisionIndex][0]
 SumSDQuality = SumSDQuality + MeanSDPrecisionOverRepetition[sc][s][r][PrecisionIndex][1]
 #print(SumMeanQuality)
 #print(SumSDQuality)
 MeanMeanQuality = SumMeanQuality / (GenParam.TotalNumberOfScenarioRepetition)
 MeanList.append(MeanMeanQuality)
 MeanSdQuality = SumSDQuality / (GenParam.TotalNumberOfScenarioRepetition)
 SdList.append(MeanSdQuality)
 AnalyseLinkFitnessFemalePrecisionEvolutionFile.write(str(sc) + "\t" +
 str(s) + "\t" +
 str(PrecisionIndex) + "\t" +
 str(MeanMeanQuality) + "\t" +
 str(MeanSdQuality) + "\n")
 AnalyseLinkFitnessFemalePrecisionEvolutionFile.close()

def AnalyseLinkReprodSuccessMaleTrait(
 ReprodSuccessByRangeByRepetitionByGenerationByAbility,
 AnalyseLinkSRMaleTraitEvolutionFile):
 MeanSDReprodMaleOverRepetition = []
 for sc in range(GenParam.Range.value):
 MeanSDReprodMaleOverRepetition.append([])
 for s in range(GenParam.TotalNumberOfSimulation):
 MeanSDReprodMaleOverRepetition[sc].append([])
 for r in range(GenParam.TotalNumberOfScenarioRepetition):
 MeanSDReprodMaleOverRepetition[sc][s].append([])
 for PrecisionIndex in range(GenParam.MaleAbilityToFindFood+1):
 MeanSDReprodMaleOverRepetition[sc][s][r].append([])
 for sc in range(GenParam.Range.value):
 for s in range(GenParam.TotalNumberOfSimulation):
 for r in range(GenParam.TotalNumberOfScenarioRepetition):
 MeanList = []
 SdList = []
 for AbilityIndex in range(GenParam.MaleAbilityToFindFood+1):
 if len(ReprodSuccessByRangeByRepetitionByGenerationByAbility[sc][r][s][0][AbilityIndex]) != 0:
 SumReprodSuccess = sum(ReprodSuccessByRangeByRepetitionByGenerationByAbility[sc][r][s][0][AbilityIndex])
 #print(len(ReprodSuccessByRangeByRepetitionByGenerationByAbility[sc][0][s][0][AbilityIndex]))
 MeanReprodSuccess = SumReprodSuccess/len(ReprodSuccessByRangeByRepetitionByGenerationByAbility[sc][r][s][0][AbilityIndex])
 #MeanList.append(MeanReprodSuccess)
 SdReprodSuccess = math.sqrt(sum([((x - MeanReprodSuccess) ** 2) for x in ReprodSuccessByRangeByRepetitionByGenerationByAbility[sc][r][s][0][AbilityIndex]]) / len(ReprodSuccessByRangeByRepetitionByGenerationByAbility[sc][r][s][0][AbilityIndex]))
 #SdList.append(SdQuality)
 MeanSDReprodMaleOverRepetition[sc][s][r][AbilityIndex].append(MeanReprodSuccess)
 MeanSDReprodMaleOverRepetition[sc][s][r][AbilityIndex].append(SdReprodSuccess)
 #print(MeanSDReprodMaleOverRepetition[sc][s][r][AbilityIndex])
 else:
 MeanSDReprodMaleOverRepetition[sc][s][r][AbilityIndex].append(0)
 MeanSDReprodMaleOverRepetition[sc][s][r][AbilityIndex].append(0)
 #print(MeanSDReprodMaleOverRepetition[sc][s][r][AbilityIndex])
 for sc in range(GenParam.Range.value):
 for s in range(GenParam.TotalNumberOfSimulation):
 MeanList = []
 SdList = []
 for AbilityIndex in range(GenParam.MaleAbilityToFindFood+1):
 SumMeanReprodSuccess = 0
 SumSdReprodSuccess = 0
 for r in range(GenParam.TotalNumberOfScenarioRepetition):
 SumMeanReprodSuccess = SumMeanReprodSuccess + \
 MeanSDReprodMaleOverRepetition[sc][s][r][AbilityIndex][0]
 SumSdReprodSuccess = SumSdReprodSuccess + \
 MeanSDReprodMaleOverRepetition[sc][s][r][AbilityIndex][1]
 #print(SumMeanReprodSuccess)
 #print(SumSdReprodSuccess)
 MeanMeanReprodSuccess = SumMeanReprodSuccess / (GenParam.TotalNumberOfScenarioRepetition)
 MeanList.append(MeanMeanReprodSuccess)
 MeanSdReprodSuccess = SumSdReprodSuccess / (GenParam.TotalNumberOfScenarioRepetition)
 SdList.append(MeanSdReprodSuccess)
 AnalyseLinkSRMaleTraitEvolutionFile.write(str(sc) + "\t" +
 str(s) + "\t" +
 str(AbilityIndex) + "\t" +
 str(MeanMeanReprodSuccess) + "\t" +
 str(MeanSdReprodSuccess) + "\n")
 AnalyseLinkSRMaleTraitEvolutionFile.close()

def AnalyseLinkReprodSuccessFemaleCriterion(
 ReprodSuccessByRangeByRepetitionByGenerationByMateChoiceCriterion,
 AnalyseLinkSRFemaleCriterionEvolutionFile):
 MeanSDReproCriterionOverRepetition = []
 for sc in range(GenParam.Range.value):
 MeanSDReproCriterionOverRepetition.append([])
 for s in range(GenParam.TotalNumberOfSimulation):
 MeanSDReproCriterionOverRepetition[sc].append([])
 for r in range(GenParam.TotalNumberOfScenarioRepetition):
 MeanSDReproCriterionOverRepetition[sc][s].append([])
 for CriterionIndex in range(GenParam.MaleAbilityToFindFood+1):
 MeanSDReproCriterionOverRepetition[sc][s][r].append([])
 for sc in range(GenParam.Range.value):
 for s in range(GenParam.TotalNumberOfSimulation):
 for r in range(GenParam.TotalNumberOfScenarioRepetition):
 MeanList = []
 SdList = []
 for CriterionIndex in range(GenParam.MaleAbilityToFindFood+1):
 if len(ReprodSuccessByRangeByRepetitionByGenerationByMateChoiceCriterion[sc][r][s][0][CriterionIndex]) != 0:
 SumReprodSuccess = sum(ReprodSuccessByRangeByRepetitionByGenerationByMateChoiceCriterion[sc][r][s][0][CriterionIndex])
 #print(len(ReprodSuccessByRangeByRepetitionByGenerationByMateChoiceCriterion[sc][0][s][0][AbilityIndex]))
 MeanReprodSuccess = SumReprodSuccess/len(ReprodSuccessByRangeByRepetitionByGenerationByMateChoiceCriterion[sc][r][s][0][CriterionIndex])
 #MeanList.append(MeanQuality)
 SdReprodSuccess = math.sqrt(sum([((x - MeanReprodSuccess) ** 2) for x in ReprodSuccessByRangeByRepetitionByGenerationByMateChoiceCriterion[sc][r][s][0][CriterionIndex]]) / len(ReprodSuccessByRangeByRepetitionByGenerationByMateChoiceCriterion[sc][r][s][0][CriterionIndex]))
 #SdList.append(SdReprodSuccess)
 MeanSDReproCriterionOverRepetition[sc][s][r][CriterionIndex].append(MeanReprodSuccess)
 MeanSDReproCriterionOverRepetition[sc][s][r][CriterionIndex].append(SdReprodSuccess)
 #print(MeanSDReproCriterionOverRepetition[sc][s][r][CriterionIndex])
 else:
 MeanSDReproCriterionOverRepetition[sc][s][r][CriterionIndex].append(0)
 MeanSDReproCriterionOverRepetition[sc][s][r][CriterionIndex].append(0)
 #print(MeanSDReproCriterionOverRepetition[sc][s][r][CriterionIndex])
 for sc in range(GenParam.Range.value):
 for s in range(GenParam.TotalNumberOfSimulation):
 MeanList = []
 SdList = []
 for CriterionIndex in range(GenParam.MaleAbilityToFindFood+1):
 SumMeanReprodSuccess = 0
 SumSdReprodSuccess = 0
 for r in range(GenParam.TotalNumberOfScenarioRepetition):
 SumMeanReprodSuccess = SumMeanReprodSuccess + MeanSDReproCriterionOverRepetition[sc][s][r][CriterionIndex][0]
 SumSdReprodSuccess = SumSdReprodSuccess + MeanSDReproCriterionOverRepetition[sc][s][r][CriterionIndex][1]
 #print(SumMeanReprodSuccess)
 #print(SumSdReprodSuccess)
 MeanMeanReprodSuccess = SumMeanReprodSuccess / (GenParam.TotalNumberOfScenarioRepetition)
 MeanList.append(MeanMeanReprodSuccess)
 MeanSdReprodSuccess = SumSdReprodSuccess / (GenParam.TotalNumberOfScenarioRepetition)
 SdList.append(MeanSdReprodSuccess)
 AnalyseLinkSRFemaleCriterionEvolutionFile.write(str(sc) + "\t" +
 str(s) + "\t" +
 str(CriterionIndex) + "\t" +
 str(MeanMeanReprodSuccess) + "\t" +
 str(MeanSdReprodSuccess) + "\n")

 AnalyseLinkSRFemaleCriterionEvolutionFile.close()

def AnalyseLinkReprodSuccessFemalePrecision(
 ReprodSuccessByRangeByRepetitionByGenerationByPrecision,
 AnalyseLinkSRFemalePrecisionEvolutionFile):

MeanSDReproPrecisionOverRepetition = []
 for sc in range(GenParam.Range.value):
 MeanSDReproPrecisionOverRepetition.append([])
 for s in range(GenParam.TotalNumberOfSimulation):
 MeanSDReproPrecisionOverRepetition[sc].append([])
 for r in range(GenParam.TotalNumberOfScenarioRepetition):
 MeanSDReproPrecisionOverRepetition[sc][s].append([])
 for PrecisionIndex in range(GenParam.TimeStepNbCompleteInformation+1):
 MeanSDReproPrecisionOverRepetition[sc][s][r].append([])
 for sc in range(GenParam.Range.value):
 for s in range(GenParam.TotalNumberOfSimulation):
 for r in range(GenParam.TotalNumberOfScenarioRepetition):
 MeanList = []
 SdList = []
 for PrecisionIndex in range(GenParam.TimeStepNbCompleteInformation+1):
 if len(ReprodSuccessByRangeByRepetitionByGenerationByPrecision[sc][r][s][0][PrecisionIndex]) != 0:
 SumReprodSuccess = sum(ReprodSuccessByRangeByRepetitionByGenerationByPrecision[sc][r][s][0][PrecisionIndex])
 #print(len(ReprodSuccessByRangeByRepetitionByGenerationByPrecision[sc][r][s][0][PrecisionIndex]))
 MeanReprodSuccess = SumReprodSuccess/len(ReprodSuccessByRangeByRepetitionByGenerationByPrecision[sc][r][s][0][PrecisionIndex])
 #MeanList.append(MeanReprodSuccess)
 SdReprodSuccess = math.sqrt(sum([((x - MeanReprodSuccess) ** 2) for x in ReprodSuccessByRangeByRepetitionByGenerationByPrecision[sc][r][s][0][PrecisionIndex]]) / len(ReprodSuccessByRangeByRepetitionByGenerationByPrecision[sc][r][s][0][PrecisionIndex]))
 #SdList.append(SdReprodSuccess)
 MeanSDReproPrecisionOverRepetition[sc][s][r][PrecisionIndex].append(MeanReprodSuccess)
 MeanSDReproPrecisionOverRepetition[sc][s][r][PrecisionIndex].append(SdReprodSuccess)
 #print(MeanSDReproPrecisionOverRepetition[sc][s][r][PrecisionIndex])
 else:
 MeanSDReproPrecisionOverRepetition[sc][s][r][PrecisionIndex].append(0)
 MeanSDReproPrecisionOverRepetition[sc][s][r][PrecisionIndex].append(0)
 #print(MeanSDReproPrecisionOverRepetition[sc][s][r][PrecisionIndex])
 for sc in range(GenParam.Range.value):
 for s in range(GenParam.TotalNumberOfSimulation):
 MeanList = []
 SdList = []
 for PrecisionIndex in range(1, GenParam.TimeStepNbCompleteInformation+1):
 SumMeanReprodSuccess = 0
 SumSdReprodSuccess = 0
 for r in range(GenParam.TotalNumberOfScenarioRepetition):
 SumMeanReprodSuccess = SumMeanReprodSuccess + MeanSDReproPrecisionOverRepetition[sc][s][r][PrecisionIndex][0]
 SumSdReprodSuccess = SumSdReprodSuccess + MeanSDReproPrecisionOverRepetition[sc][s][r][PrecisionIndex][1]
 #print(SumMeanReprodSuccess)
 #print(SumSdReprodSuccess)
 MeanMeanReprodSuccess = SumMeanReprodSuccess / (GenParam.TotalNumberOfScenarioRepetition)
 MeanList.append(MeanMeanReprodSuccess)
 MeanSdReprodSuccess = SumSdReprodSuccess / (GenParam.TotalNumberOfScenarioRepetition)
 SdList.append(MeanSdReprodSuccess)
 AnalyseLinkSRFemalePrecisionEvolutionFile.write(str(sc) + "\t" +
 str(s) + "\t" +
 str(PrecisionIndex) + "\t" +
 str(MeanMeanReprodSuccess) + "\t" +
 str(MeanSdReprodSuccess) + "\n")

 AnalyseLinkSRFemalePrecisionEvolutionFile.close()
